# Multiple imputation with missing indicators as proxies for unmeasured variables: simulation study

**DOI:** 10.1186/s12874-020-01068-x

**Published:** 2020-07-08

**Authors:** Matthew Sperrin, Glen P. Martin

**Affiliations:** Faculty of Biology, Medicine and Health, Vaughan House, University of Manchester, Manchester, M13 9PL UK

**Keywords:** Missing data, Missing indicator, Multiple imputation, Simulation study

## Abstract

**Background:**

Within routinely collected health data, missing data for an individual might provide useful information in itself. This occurs, for example, in the case of electronic health records, where the presence or absence of data is informative. While the naive use of missing indicators to try to exploit such information can introduce bias, its use in conjunction with multiple imputation may unlock the potential value of missingness to reduce bias in causal effect estimation, particularly in missing not at random scenarios and where missingness might be associated with unmeasured confounders.

**Methods:**

We conducted a simulation study to determine when the use of a missing indicator, combined with multiple imputation, would reduce bias for causal effect estimation, under a range of scenarios including unmeasured variables, missing not at random, and missing at random mechanisms. We use directed acyclic graphs and structural models to elucidate a variety of causal structures of interest. We handled missing data using complete case analysis, and multiple imputation with and without missing indicator terms.

**Results:**

We find that multiple imputation combined with a missing indicator gives minimal bias for causal effect estimation in most scenarios. In particular the approach: 1) does not introduce bias in missing (completely) at random scenarios; 2) reduces bias in missing not at random scenarios where the missing mechanism depends on the missing variable itself; and 3) may reduce or increase bias when unmeasured confounding is present.

**Conclusion:**

In the presence of missing data, careful use of missing indicators, combined with multiple imputation, can improve causal effect estimation when missingness is informative, and is not detrimental when missingness is at random.

## Background

Missing data is a common feature in observational studies. It is conventional to view missing data as a nuisance, and as such, methods to handle missing data usually target an estimand that would be available in the absence of missing data (completed data estimand). The mechanism for missingness is conventionally divided into three categories: missing completely at random (MCAR), missing at random (MAR), and missing not at random (MNAR) [1, 2]. In the case of MCAR and MAR, an unbiased estimator of any completed data estimand exists. Such an estimator is provided by complete case analysis in MCAR scenarios, and by multiple imputation in both MAR and MCAR scenarios. In contrast, under MNAR, unbiased estimators of a given completed data estimand may or may not exist, depending on the nature of the estimand, and the joint distribution of the missingness mechanism and the other variables under consideration [2].

Alongside missing data, confounding is a threat to causal effect estimation in observational studies, especially where this is caused by unmeasured variables. Where unmeasured confounding exists, it is not possible to construct unbiased estimators of a causal effect, without making strong, unverifiable assumptions [3].

For example, consider a scenario in which we are interested in calculating the causal effect of total cholesterol (exposure) on cardiovascular disease (outcome), using electronic health records. Presence (analogous to missingness) of a cholesterol test result for a particular patient indicates that a decision was made to run this test, and the reason for this decision is likely to depend on characteristics of the patient; for example the patient’s diet, which may or may not be recorded. Diet may affect both the result of the laboratory test, and the outcome of interest, hence confounding. If information concerning diet is not recorded, we therefore have unmeasured confounding, and unbiased estimators of the causal estimand may not exist. Moreover, the missingness mechanism for the exposure may depend on unmeasured variables, in which case the exposure is MNAR, and an unbiased estimator of the completed data estimand may not exist either.

An emerging hypothesis is that in scenarios such as this, missing data may be a blessing rather than a curse, because the missingness mechanism can be used as a proxy for the unmeasured confounding, through the use of missing indicators [4]. Suppose one wished to use missing data approaches to target the causal estimand directly (rather than the completed data estimand, as is done conventionally [5]). Then, exploiting the missingness mechanism through the use of missing indicators may reduce bias even compared with estimation in the absence of missing data, particularly when the missing indicator is used in conjunction with multiple imputation (MIMI) [4, 6–8]. This is despite that naïve use of missing indicators introduces bias in the completed data estimand under MAR and MCAR [9–12].

In this paper we investigate, through simulation supplemented with analytical findings, the potential for using the missingness mechanism to partly adjust for unmeasured confounding and other missing not at random scenarios, and identify the cases where this can reduce bias for causal effect estimation.

## Methods

### Scenarios and data generating mechanisms

**Fig. 1:**
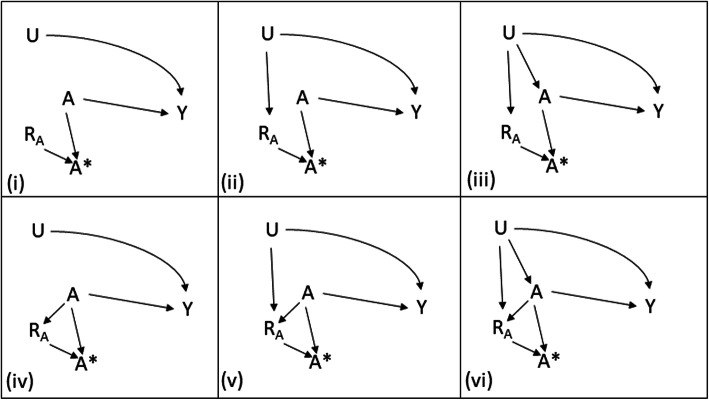
Causal directed acyclic graphs denoting missingness mechanism for A, R_A_: six scenarios considered in the paper

Our aim is to identify missing data strategies that recover causal effects of an exposure on an outcome, with minimal bias in a variety of scenarios, especially where the causal effects are affected by unmeasured confounding. The scenarios that we consider in this paper are given in Fig. 1. We consider a partially observed exposure *A*, a fully observed outcome *Y* and a further variable *U*, which is either fully unobserved or fully observed depending on the mechanism for missingness. The missingness indicator for *A* is *R*_*A*_ where *R*_*A*_ = 0 when *A* is missing. In the example in the Introduction, *A* is total cholesterol, *Y* is cardiovascular disease, *U* is diet, and *R*_*A*_ denotes whether a cholesterol test has been performed or not. *A*^∗^ is the observable part of *A*, i.e. *A*^∗^ = *A* when *R*_*A*_ = 1, and missing when *R*_*A*_ = 0. So *A*^∗^ is what we observe, while *A* includes unobserved values.

We use the counterfactual notation for consideration of causal effects, e.g. *Y*(*A* = *a*) denotes the value of *Y* that would be observed if, possibly contrary to fact, we set *A* = *a*, and we will abbreviate to *Y*(*a*) where this does not lead to ambiguity. See [3] for an introduction to causal inference with counterfactuals. Our primary aim is to recover the unconditional causal effect of *A* on *Y*; for continuous exposure, *A*, that is the expected effect on *Y* for 1-unit increase in *A*: *δ*_*A*_ ≔ *E*[*Y*(*A* = *a* + 1) − *Y*(*A* = *a*)]. We also have a secondary interest in inferring the presence of unmeasured confounding (i.e. whether an unobserved *U* directly affects both *A* and *Y*) or missing not at random mechanisms, or both.

First, we consider scenarios in Fig. 1 where *U* is assumed to be unobserved, which we label (i)-(vi). Scenario (i) corresponds to MCAR, since *R*_*A*_ is independent of all other variables. All other scenarios, (ii)-(vi), are MNAR since *R*_*A*_ is dependent on *U* or *A* or both. Note here that we follow the graphical definitions of MCAR, MAR and MNAR as set out in [2]. In scenarios (i) and (iv), complete case analysis can yield unbiased estimates of the causal effect of *A* on *Y* (see e.g. [13] for scenario (iv)). In scenarios (iii) and (vi), the unobserved variable *U* confounds the relationship between *A* and *Y*, so an unbiased estimate of the causal effect of *A* on *Y* may not be available even if there were no missingness.

In scenarios (ii), (iii), (v), and (vi), we could view *R*_*A*_ as a proxy for the unobserved *U*. It therefore may be beneficial to include *R*_*A*_ in the outcome model. This may reduce bias in the estimation of the causal effect of *A* on *Y*, by partly adjusting for the confounding effect of *U*.

Second, we consider each of the six scenarios in Fig. 1 with *U* assumed fully observed, and label these (i-U)-(vi-U). Here, we do not expect any benefit in including *R*_*A*_ in the outcome model, but we wish to examine any reduction in performance that doing so may introduce. Scenario (i-U) remains MCAR, while scenarios (ii-U) and (iii-U) are now MAR. Scenarios (iv-U), (v-U), and (vi-U) remain MNAR but only through the dependence of *R*_*A*_ on *A*.

We now specify the structural models that will be assumed for our further derivations and simulations.

- *U* is binary with *P*[*U* = 1] = *π*_*U*_.
- *A* is continuous, with $A\sim N\left({\alpha}_0+{\alpha}_UU,{\sigma}_A^2\right)$.
- *R*_*A*_ is binary, with either *P*[*R*_*A*_ = 0] = expit(*β*_0_ + *β*_*U*_*U* + *β*_*A*_*A* + *β*_*UA*_*UA*), or simply *R*_*A*_ = 1 − *U*, depending on the scenario considered.
- *Y* is continuous, with $Y\sim N\left({\gamma}_0+{\gamma}_UU+{\gamma}_AA+{\gamma}_{UA} UA,{\sigma}_Y^2\right)$.

The outcome model is linear in *A* hence the causal effect of a one-unit change in *A* does not depend on the starting value of *A*, however it does depend on *U* because of the interaction term. Specifically, the (true) unconditional causal effect of interest, by standardization, is *δ*_*A*_ = *E*[*Y*(*A* = *a* + 1) − *Y*(*A* = *a*)] = *γ*_*A*_ + *π*_*U*_*γ*_*UA*_.

### Considered approaches

For notation, we use Greek letters with no superscripts to denote true parameter values (from the data generating mechanisms described in the previous section) – e.g. *γ*_*A*_ - and use the same Greek letters with bracketed superscripts to denote the parameters estimated in the various analysis models – e.g. ${\gamma}_A^{(1)}$. We consider the following imputation and modelling approaches.

First, a complete case analysis. When *U* is unobserved, this fits the model $E\left[Y\right]={\gamma}_0^{(0)}+{\gamma}_A^{(0)}{A}^{\ast }$, restricting to observations where *R*_*A*_ = 1. When *U* is observed, the model is $E\left[Y\right]={\gamma}_0^{(0U)}+{\gamma}_A^{(0U)}{A}^{\ast }+{\gamma}_U^{(0U)}U+{\gamma}_{UA}^{(0U)}U{A}^{\ast }$.

Second, we consider multiple imputation, under a joint normal model assuming a MAR mechanism. Thus, when *U* is unobserved the imputation model is $E\left[A\right]={\phi}_0^{(I)}+{\phi}_Y^{(I)}Y$, and when *U* is observed the imputation model is $E\left[A\right]={\phi}_0^{(IU)}+{\phi}_Y^{(IU)}Y+{\phi}_U^{(IU)}U+{\phi}_{UY}^{(IU)} UY$ (including the interaction term, as recommended in [14]). Missing *A* s are imputed by a random draw from the predictive distribution implied by the imputation model, using ‘proper’ imputation which accounts for both the uncertainty in the imputation model, and the residual variance [15]. This is repeated multiple times and subsequent results are pooled over iterations using Rubin’s rules (in this study we consider five imputations for the sake of computational time).

Throughout, we denote the imputed *A* as *A*_imp_. We then consider the following three outcome/analysis models, when *U* is unobserved:

1. ‘MI(A)’: $$ E\left[Y\right]={\gamma}_0^{(1)}+{\upgamma}_A^{(1)}{A}_{\mathrm{imp}} $$.
2. ‘MI(R + A)’: $$ E\left[Y\right]={\gamma}_0^{(2)}+{\gamma}_A^{(2)}{A}_{\mathrm{imp}}+{\gamma}_R^{(2)}\left(1-{R}_A\right) $$.
3. ‘MI(R*A)’: $$ E\left[Y\right]={\gamma}_0^{(3)}+{\gamma}_A^{(3)}{A}_{\mathrm{imp}}+{\gamma}_R^{(3)}{R}_A+{\gamma}_{RA}^{(3)}{A}_{\mathrm{imp}}\left(1-{R}_A\right) $$.

Model 1 represents a standard multiple imputation (MI) approach, while models 2 and 3 are variants of the MIMI approach, without and with interaction (MI(R + A), MI(R*A)).

When *U* is observed we consider three outcome models of the same form:

1. U. ‘MI(A)’: $$ E\left[Y\right]={\gamma}_0^{(1U)}+{\upgamma}_A^{(1U)}{A}_{\mathrm{imp}}+{\upgamma}_U^{(1U)}U++{\upgamma}_{UA}^{(1U)}U{A}_{\mathrm{imp}} $$.
2. U. ‘MI(R + A)’: $$ E\left[Y\right]={\gamma}_0^{(2U)}+{\gamma}_A^{(2U)}{A}_{\mathrm{imp}}+{\gamma}_R^{(2U)}\left(1-{R}_A\right)+{\upgamma}_U^{(2U)}U++{\upgamma}_{UA}^{(2U)}U{A}_{\mathrm{imp}} $$.
3. U. ‘MI(R*A)’: $$ E\left[Y\right]={\gamma}_0^{(3U)}+{\gamma}_A^{(3U)}{A}_{\mathrm{imp}}+{\gamma}_R^{(3U)}{R}_A+{\gamma}_{RA}^{(3U)}{A}_{\mathrm{imp}}\left(1-{R}_A\right)+{\upgamma}_U^{(3U)}U++{\upgamma}_{UA}^{(3U)}U{A}_{\mathrm{imp}} $$.

Finally, we also include ‘completed data’ models in which we use the original variable *A* in our models. This serves as a ground-truth for all analyses in the absence of missing data. In scenarios (i)-(vi), *U* is not observed hence the completed data model is $E\left[Y\right]={\gamma}_0^{(C)}+{\gamma}_A^{(C)}A$, while in scenarios (i-U)-(vi-U) *U* is observed, hence $E\left[Y\right]={\gamma}_0^{(CU)}+{\gamma}_A^{(CU)}A+{\gamma}_U^{(CU)}U+{\gamma}_{UA}^{(CU)} UA$.

When *U* is not observed, by standardisation we would hope that $E\left[{\hat{\gamma}}_A^{(j)}\right]\approx {\delta}_A={\gamma}_A+{\gamma}_{UA}{\pi}_U$ (for *j* = 0, 1, 2, *C*), which represents the unconditional causal effect of *A* on *Y*. In MI(R*A), the (1 − *R*_*A*_) term may act as a partial proxy for *U*, therefore inclusion of the interaction means we expect ${\hat{E\Big[\gamma}}_A^{(3)}\Big]$ to lie between the unconditional and marginal causal effects of *A* on *Y*.

For the cases where *U* is observed, we hope that $E\left[{\hat{\gamma}}_A^{(j)}\right]\approx {\gamma}_A$ for all models (*j* = 0*U*, 1*U*, 2*U*, 3*U*, *CU*), since the interaction with *U* is always modelled.

Where present in our models, we hypothesise that the ${\hat{\gamma}}_R^{(j)}$ and ${\hat{\gamma}}_{RA}^{(j)}$ terms may indicate MNAR when they are nonzero.

### Analytical comments

It is instructive to consider a special case of scenario (ii) (see Fig. 1), in which *R*_*A*_ = 1 − *U*. Suppose further that the true underlying regression model has no interaction, i.e. *E*[*Y*] = *γ*_0_ + *γ*_*A*_*A* + *γ*_*U*_*U*. Performing multiple imputation for *A* and including a missing indicator 1 − *R*_*A*_ in the outcome model – which corresponds to the MI(R + A) approach described above (model 2) - would be expected to perform well, as this analysis model matches the true model. Indeed, the regression coefficient of *A* on *Y* can be estimated without bias, $E\left[{\hat{\gamma}}_A^{(2)}\right]$ = $E\left[{\hat{\gamma}}_A\right]$ = *γ*_*A*_. However, the model produces a biased estimate of the regression coefficient of *Y* on *U*, $E\left[{\hat{\gamma}}_R^{(2)}\right]=E\left[{\hat{\gamma}}_U\right]\approx {\gamma}_U\frac{\sigma_Y^2}{\gamma_A^2{\sigma}_A^2+{\sigma}_Y^2}$. This is because fitting the imputation model introduces regression dilution [16]. A justification is given in the Appendix. We emphasise that this should not be viewed as a shortcoming of multiple imputation, since multiple imputation in this case is targeting the regression coefficient of *Y* on *A* in the absence of missing data (completed data estimand), *γ*_*A*_.

While the case *R*_*A*_ = 1 − *U* may seem extreme, it could approximately hold in practice: for example, if a particular test (*A*) is commonly run if a particular unrecorded condition (*U*) is met, and is rarely run otherwise.

### Simulation study set-up

The aims, general structure, and models, are described above. We consider the following specific data generating mechanisms, which cover all of the scenarios (i)-(vi) and (i-U)-(vi-U) described in Fig. 1. We closely follow best practice for the design and reporting of simulation studies as proposed in [17].

For the *R*_*A*_ ≠ 1 − *U* case:

- We fix the sample size (number of observations within each simulation run) to be *n* = 10,000, and fix *π*_*U*_ = 0.5.
- We choose the intercepts as functions of the other parameters: *α*_0_ such that *E*[*A*] = 0, *γ*_0_ such that *E*[*Y*] = 0, and *β*_0_ such that *P*[*R*_*A*_ = 0] varies over the grid {0.25,0.5,0.75}.
- The main effect parameters, *α*_*U*_, *β*_*A*_, and *γ*_*U*_ are all varied over the grid {0,0.1,0.5,1}, the parameter *β*_*U*_ over the grid {−1, 0, 0.1,0.5,1} (a negative *β*_*U*_ is included to study whether the direction of correlation between *U* and *R*_*A*_ is important), while we fix *γ*_*A*_ = 1.
- The interaction effect parameters, *β*_*UA*_ and *γ*_*UA*_, are varied between {0,0.5}.
- The standard deviation of *Y*, *σ*_*Y*_, is varied over the grid {0.1,0.5,1}, while we fix *σ*_*A*_ = 1.

For the *R*_*A*_ = 1 − *U* case, we use the same simulation settings with the following exceptions:

- We exclude *β*_*U*_, *β*_*A*_ and *β*_*UA*_, which are redundant.
- We vary *π*_*U*_ over the grid {0.25,0.5,0.75}, as this is required to vary the proportion of missingness.

All combinations of the parameters are evaluated, resulting in 11,808 scenarios, of which 288 cover the case where *R*_*A*_ = 1 − *U*.

For each scenario, we fit the models described in the previous section, and report estimates of the outcome coefficients from the various models. Each scenario is repeated 200 times and summary statistics over these iterations retained. For all parameters of interest – those of the form ${\hat{\gamma}}_A^{(j)}$, ${\hat{\gamma}}_R^{(j)}$, and ${\hat{\gamma}}_{RA}^{(j)}$, we retain the 2.5th, 25th, 50th, 75th and 97.5th percentile parameter estimates. We also retain the average model-based standard errors and empirical standard errors for each parameter. For the parameters ${\hat{\gamma}}_A^{(j)}$ we also report the length and coverage of associated confidence intervals.

## Results

Here we present a subset of the simulations that capture the main findings; full results are available – see *Availability of data and materials*. Throughout this section we restrict parameters to *γ*_*A*_ = 1, *γ*_*U*_ = 1, and *σ*_*Y*_ = 1, although we consider both *γ*_*UA*_ = 0 and *γ*_*UA*_ = 0.5. We also restrict to cases that result in *P*[*R*_*A*_ = 1] = 0.5. When *γ*_*UA*_ = 0, the marginal causal effect of *A* on *Y*, and the conditional causal effect of *A* on *Y* (when *U* = 0 and when *U* = 1) are 1. In this case, complete case analysis agrees closely with the completed data estimates. Most of our results focus on this case, for simplicity. When *γ*_*UA*_ = 0.5, by standardisation the marginal causal effect of *A* on *Y*, throughout, is 1.25, while the conditional causal effects of *A* on *Y* given *U* = 0 and *U* = 1 are 1 and 1.5 respectively. Qualitatively similar results were found when varying the remaining parameters in the outcome model and proportion of missing data. Results are summarized in figures and tables. The tables include mean estimates, average confidence interval width, and coverage for a targeted value of 1 in all cases.

**Fig. 2:**
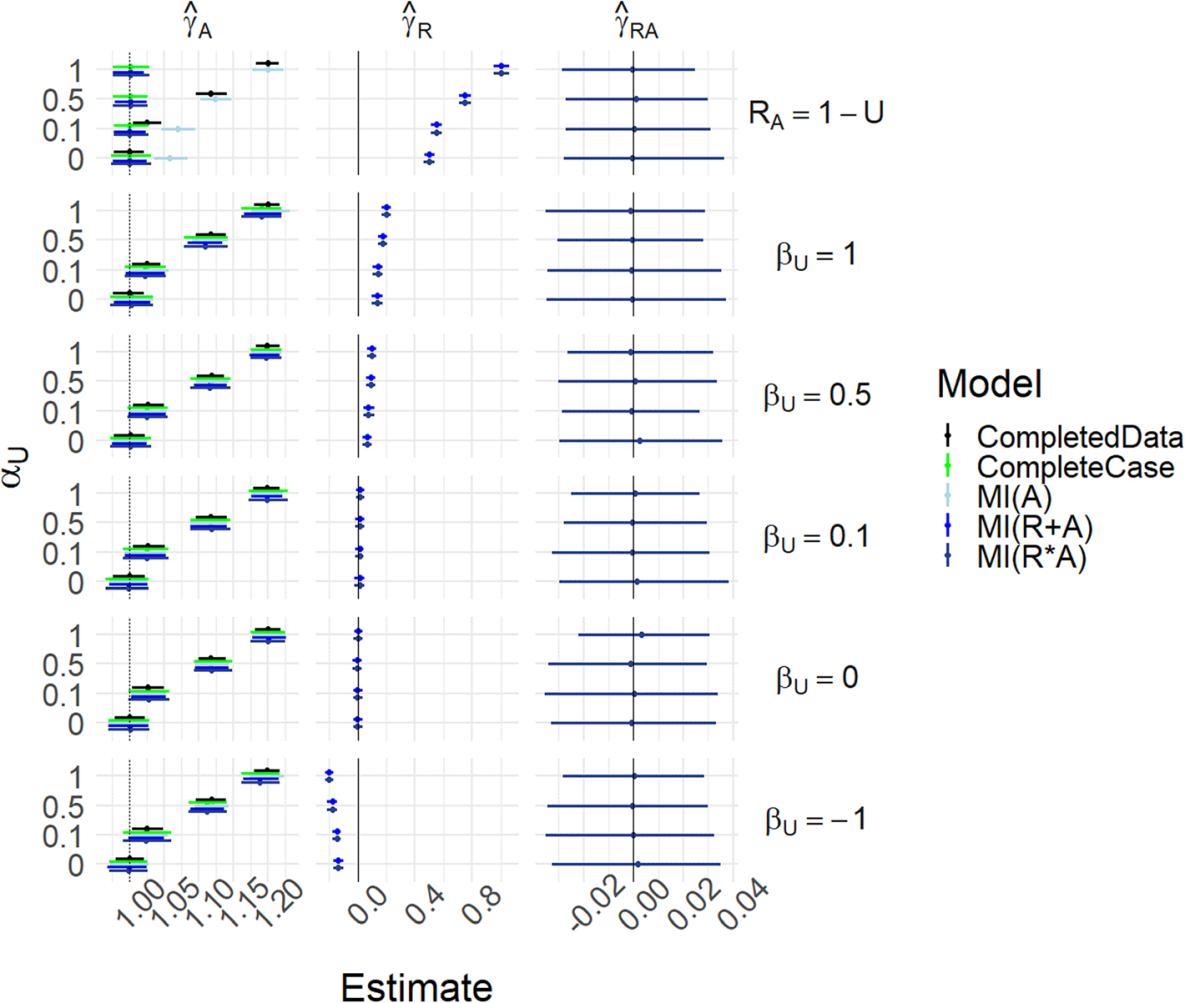
Results for scenarios (i)-(iii), with γ_UA_ = 0. Mean of estimated coefficients across simulations; error bars represent the 2.5th and 97.5th percentiles. Columns are different parameter estimates, rows are different values of β_U_, with the special case R_A_ = 1 − U on the top row. Within each graph, the y-axis varies α_U_

Figure 2 and Table S1 show results for Scenarios (i)-(iii), i.e. where *β*_*A*_ = *β*_*UA*_ = 0. In addition, for this figure, we fix *P*[*R*_*A*_ = 1] = 0.5, *γ*_*A*_ = 1, *γ*_*U*_ = 1, *γ*_*UA*_ = 0 and *σ*_*A*_ = 1. Scenario (i) is the case where *β*_*U*_ = *α*_*U*_ = 0. For Scenario (ii), *β*_*U*_ controls the strength of the relationship between *U* and *R*_*A*_, with the extreme case *R*_*A*_ = 1 − *U*, with *α*_*U*_ = 0. For Scenario (iii), *α*_*U*_ additionally controls the strength of the relationship between *U* and *A* – i.e. introduces unmeasured confounding.

The causal effect of *A* on *Y* is 1 (dotted vertical line in leftmost panels). For *β*_*U*_ = *α*_*U*_ = 0 all methods’ estimates of *γ*_*A*_ are able to recover this without bias and with appropriate coverage. As *β*_*U*_ increases, all methods are still able to estimate the causal effect well, except that MI(A) becomes biased when *R*_*A*_ = 1 − *U*. As *α*_*U*_ increases, the completed data model becomes biased because of unmeasured confounding. We see that the MIMI approaches and complete case analysis are able to mitigate this to some extent, and successfully when *R*_*A*_ = 1 − *U*. The estimate of *γ*_*R*_ becomes nonzero for the MIMI methods when *α*_*U*_ ≠ 0: it is through this that the MIMI methods are able to partly correct for the unmeasured confounding. Note that when *β*_*U*_ =  − 1 then the ${\hat{\gamma}}_R^{(j)}$ s are negative rather than positive, but the ${\hat{\gamma}}_A^{(j)}$ s are similar to the *β*_*U*_ = 1 case.

**Fig. 3:**
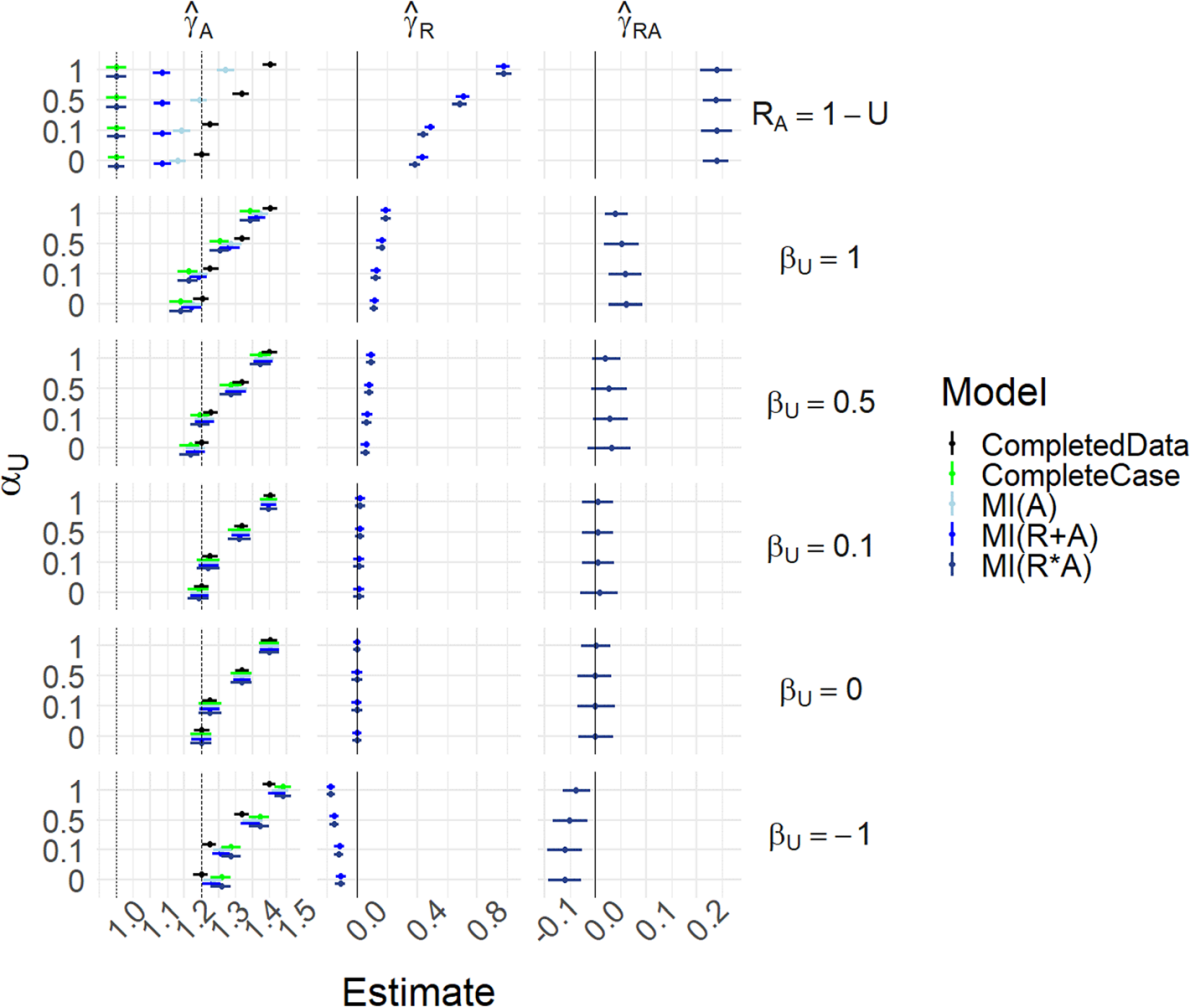
Results for scenarios (i)-(iii), with γ_UA_ = 0.5. Mean of estimated coefficients across simulations; error bars represent the 2.5th and 97.5th percentiles. Columns are different parameter estimates, rows are different values of β_U_, with the special case R_A_ = 1 − U on the top row. Within each graph, the y-axis varies α_U_

Figure 3 and Table S2 present the same scenarios as Fig. 2 but with *γ*_*UA*_ = 0.5; hence the marginal and conditional causal effects of *A* on *Y* differ, as explained above. In the *R*_*A*_ = 1 − *U* case, with *α*_*U*_ = 0 the completed data model estimates *δ*_*A*_ = *γ*_*A*_ + *π*_*U*_*γ*_*UA*_ = 1.25, the marginal effect, while complete case analysis estimates the conditional effect when *U* = 0 (which is *γ*_*A*_ = 1); this is of course not surprising as there is only data when *U* = 0. The MI(R*A) estimate agrees with the complete case, while MI(A) and MI(R + A) tend to interpolate between the two. However, when *β*_*U*_ =  − 1 we now see that all methods have increased bias. This is because the reversal of the correlation between *U* and *R*_*A*_ means that missingness is more likely when *U* = 1, when the conditional causal effect is 1.5.

**Fig. 4:**
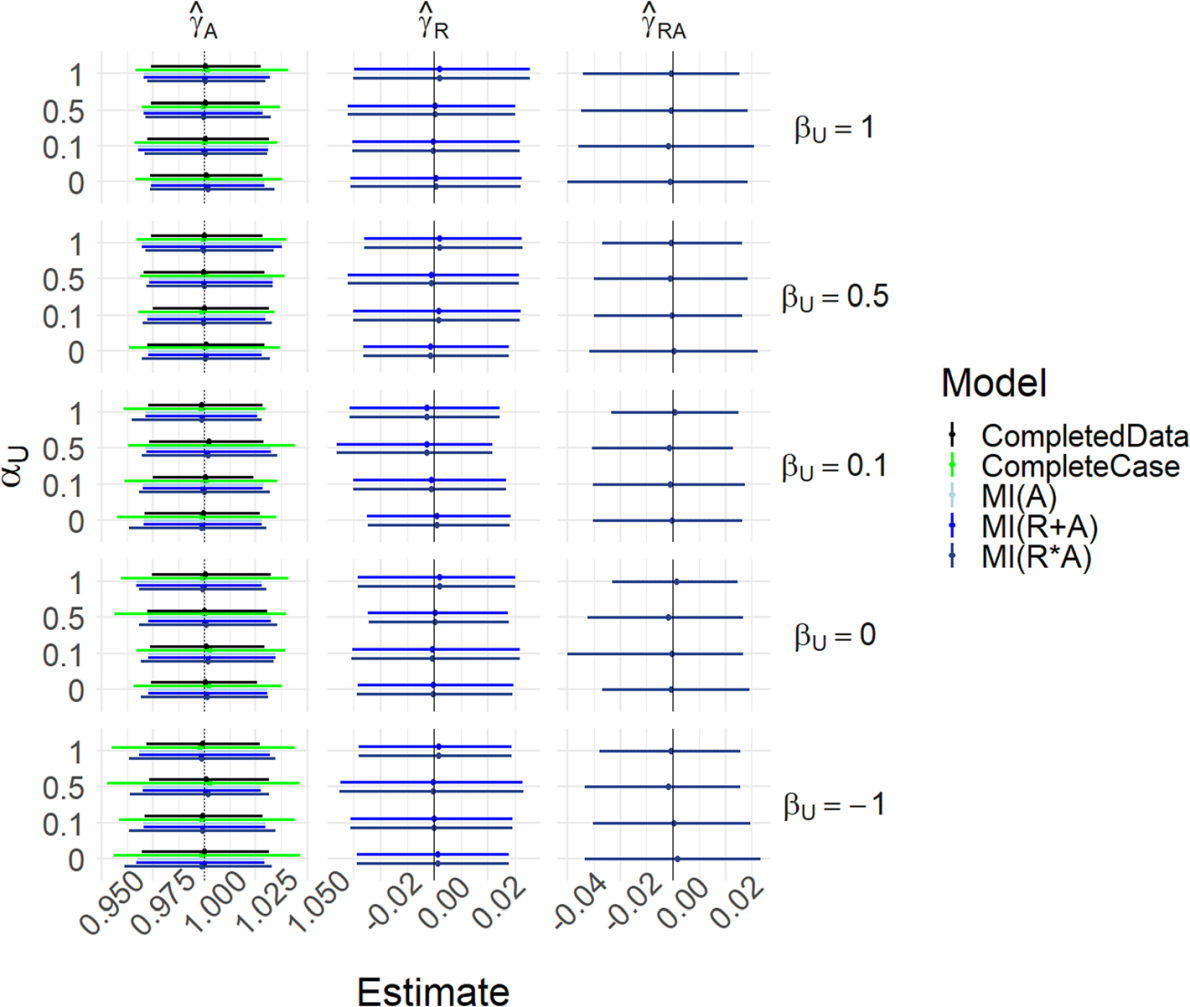
Results for scenarios (i-U)-(iii-U), with γ_UA_ = 0. Mean of estimated coefficients across simulations; error bars represent the 2.5th and 97.5th percentiles. Columns are different parameter estimates, rows are different values of β_U_, with the special case R_A_ = 1 − U on the top row. Within each graph, the y-axis varies α_U_

Figure 4 and Table S3 show results for scenario (i-U)-(iii-U) with *γ*_*UA*_ = 0, i.e. the same conditions as Fig. 2 except that *U* is now measured. In this MAR case, the missing indicator should be redundant, and the concern is that its inclusion may introduce bias into the estimates. We see that all methods perform well in recovering *γ*_*A*_. MI(A) and MI(R + A) are in almost perfect agreement.

**Fig. 5:**
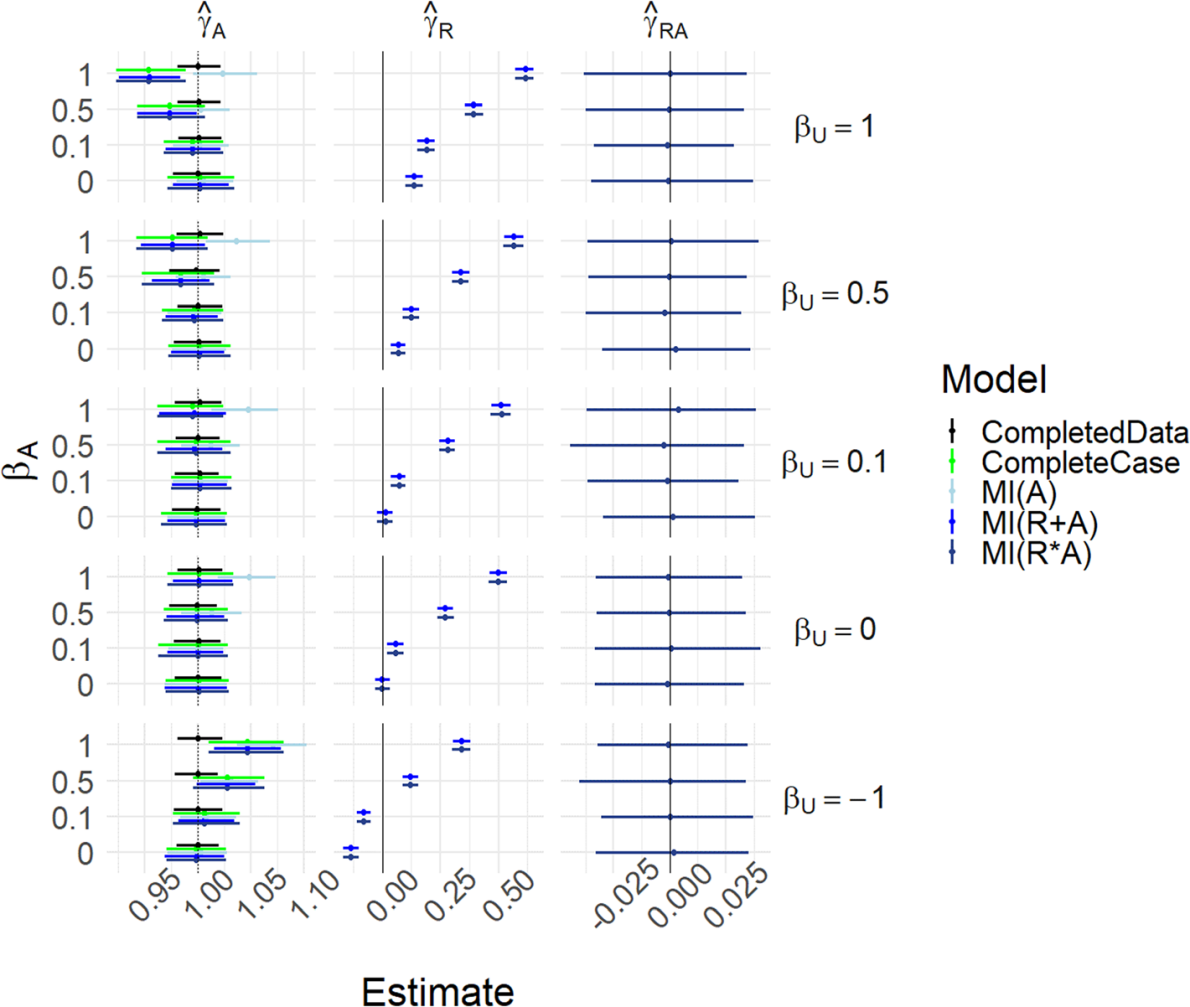
Results for scenarios (iv) and (v), with γ_UA_ = 0. Mean of estimated coefficients across simulations; error bars represent the 2.5th and 97.5th percentiles. Columns are different parameter estimates, rows are different values of β_U_. Within each graph, the y-axis varies β_A_

Figure 5 and Table S4 show results for scenarios (iv) and (v), where we examine the effects of missingness in *A* depending on *A* itself. Here, the scenario dictates that *α*_*U*_ = *β*_*UA*_ = 0, and we additionally fix *γ*_*A*_ = 1, *γ*_*U*_ = 1, *γ*_*UA*_ = 0, *σ*_*Y*_ = 1 and *σ*_*A*_ = 1. The key varying parameters are *β*_*A*_, which controls the dependence of *R*_*A*_ on *A*, and *β*_*U*_, which controls the dependence of *R*_*A*_ on *U*.

When *β*_*U*_ = 0 (corresponding to scenario (iv)), MI(A) is biased in estimating *γ*_*A*_. However, MI(R + A) and MI(R*A) are not biased. When *β*_*U*_ ≠ 0 things are more complicated, and there is no clear approach that minimizes the bias. What is consistent, however, is that *γ*_*R*_ estimates are nonzero when either *β*_*U*_ or *β*_*A*_ are not zero.

**Fig. 6:**
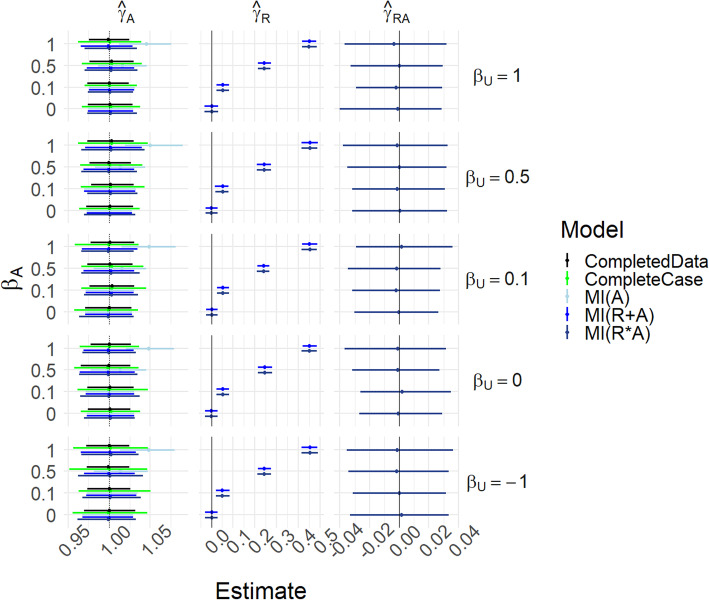
Results for scenarios (iv-U) and (v-U), with γ_UA_ = 0. Mean of estimated coefficients across simulations; error bars represent the 2.5th and 97.5th percentiles. Columns are different parameter estimates, rows are different values of β_U_. Within each graph, the y-axis varies β_A_

Figure 6 and Table S5 show results for Scenario (iv-U) and (v-U), which are the same scenarios as in Fig. 5 except that *U* is measured. In these cases, changing values of *β*_*U*_ do not cause particular problem for any method, while nonzero *β*_*A*_ introduces bias in estimation of *γ*_*A*_ for MI(A) only.

**Fig. 7:**
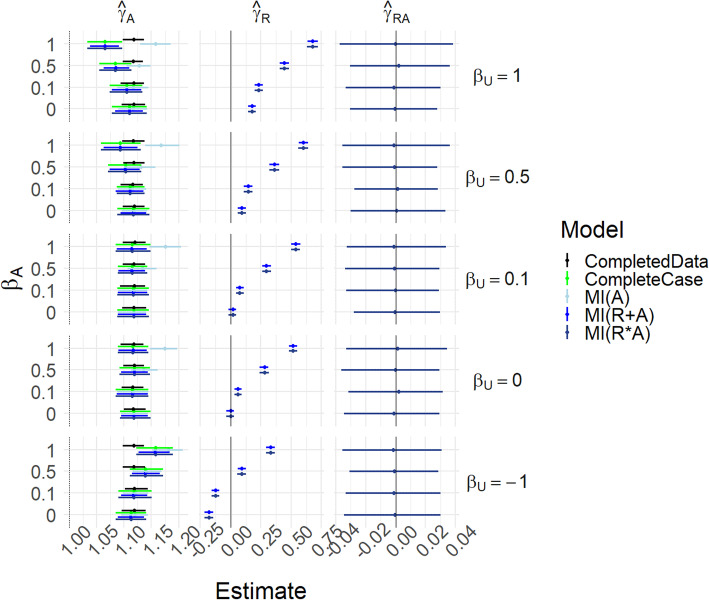
Results for scenario (vi), with γ_UA_ = 0 and α_U_ = 0.5. Mean of estimated coefficients across simulations; error bars represent the 2.5th and 97.5th percentiles. Columns are different parameter estimates, rows are different values of β_U_. Within each graph, the y-axis varies β_A_

Figure 7 and Table S6 show results for Scenario (vi). This is the most flexible scenario with no constraints on the parameter values. Here we illustrate the case where *γ*_*A*_ = 1, *γ*_*U*_ = 1, *γ*_*UA*_ = 0.5, *σ*_*A*_ = 1, *σ*_*Y*_ = 1 and *β*_*UA*_ = 0, and *α*_*U*_ = 0.5.

The results are similar to those for Scenario (v) except that *γ*_*A*_ is more commonly overestimated.

Further results are given in the Supplements: Figs. S1-S3 and corresponding Tables S7-S9.

## Discussion

In this paper we have explored, through simulation, the potential merits of supplementing multiple imputation with a missing indicator, particularly in circumstances where missingness is not at random, and the missingness may moreover act as a proxy for unmeasured confounding. We emphasise that, in contrast to the usual missing data literature that targets completed data estimands, here we target causal estimands that are not available in general even with completed data (because of unmeasured confounding). In scenarios where missingness of an exposure depends on an unmeasured confounder, the missingness indicator can be used as a proxy for the unmeasured confounding, and this may reduce bias in some situations. Careful consideration of the likely missingness mechanisms for a given clinical question/ dataset is key to deciding on the analytical approach.

In the MCAR and MAR scenarios, without unmeasured confounding, adding a missing indicator to multiple imputation did not introduce bias in estimation of causal effects. In the MNAR scenarios without unmeasured confounding, adding a missing indicator generally reduced bias compared with multiple imputation alone. In the presence of unmeasured confounding, bias in estimation was sometimes better and sometimes worse when including a missing indicator and/or its interaction with the main effect, depending on the relationships between the parameters. This reflects the potentially complex relationships, and shows that care should be given, and decisions based on a study-by-study basis. In all cases, when unmeasured confounding and/or MNAR exists, the missing indicator coefficient and/or its interaction with the main effect coefficient were estimated to be non-zero. These non-zero effect estimates of the missing indicators act as a signal that there may be MNAR mechanisms present, and hence it would be difficult or impossible to obtain unbiased causal effects. Any disagreement between the main effect parameter estimates with and without including a missing indicator provide a similar indication.

The ‘missing indicator’ approach has a somewhat negative reputation in the causal inference literature. This is because it is usually coupled with a weak approach to impute the missing data itself - such as using the unconditional mean [8]. With such application, missing indicator is known to lead to biased estimation even under MCAR [9–12]. The idea of combining the missing indicator approach with multiple imputation was first proposed by [6], and has been further explored by [4, 7]. In those articles, the focus is on handling missing data in covariates used in propensity scores, whereas here we consider missing data in the exposure of interest. Nevertheless, [4] in particular noted that the use of missing indicators can partly adjust for unmeasured confounding, similar to our findings. However, we find that they can also make the situation worse, so considerable care is needed, on a study-by-study basis.

### Strengths and limitations

We explored a wide range of simulation settings in a fully factorial design. While we can only present a limited range of results in the paper, the simulation code and results are available online for inspection. Nevertheless, simulations are necessarily simpler than scenarios that might be encountered in practice. First, missingness may affect many covariates. While addition of missing indicators, and interactions, seems robust, it may break down in some scenarios with complex multivariate patterns of missingness, and may also lead to unacceptable model complexity. Second, there may be multiple unmeasured or partially measured confounders. However, we could consider multiple confounders as being summarized by a propensity score, for example, and thus we expect the results here to generalize to the multiple confounders case. We emphasise that we focused on missing data in exposure where the causal estimand rather than the completed data estimand was targeted, and that results here should not be generalized to different scenarios [18]. Finally while we have presented limited analytical findings in this paper, it is likely that the bias formulas could be derived for a wide range of the scenarios presented, which we leave as a topic for further research.

## Conclusions

We recommend that addition of a missing indicator, and corresponding interaction terms, can supplement, but not replace, standard multiple imputation. In particular, we recommend the use of MIMI (including interactions between missing indicators and the corresponding variable) as a strategy for handling missing data in causal effect estimation problems. Non-zero estimates of the missing indicator then alert to possible occurrence of MNAR and/or unmeasured confounding, and the need for further sensitivity analysis. We caveat that the use of missing indicators should not replace careful consideration of assumed plausible causal structures, and drawing a causal diagram to depict these assumptions remains the starting point for a well-conducted causal inference.

### Supplementary information

**Additional file 1.**

## Data Availability

All simulation results are available at https://figshare.com/articles/Output_from_simulations/10320617, and the code is available at https://github.com/mattsperrin/missing_indicator_sim_paper.

## Appendix

### Bias for estimating *γ*_*u*_ from imputed data when *R*_*A*_ = 1 − *U*

Here we give an informal justification for the bias result. In this section we use the superscript ∗ to denote true values of parameters.

First consider the imputation model

$$ {a}_i={\phi}_0^{\ast }+{\phi}_Y^{\ast }{y}_i+{\delta}_i, $$

for $\mathcal{J}=\left\{i:{R}_{A,i}=1\right\}$, i.e. non-missing *A* s, with *δ*_*i*_ ∼ *N*(0, *τ*^2^). Note that *u*_*i*_ does not appear in this model because *u*_*i*_ = 0 for all $i\in \mathcal{J}$.

Now ${y}_i\sim N\left({\mu}_{Y,i}={\gamma}_0^{\ast }+{\gamma}_A^{\ast }{a}_i,{\sigma}_Y^2\right)$ for $i\in \mathcal{J}$. In analogy with p175 of [16], consider a hypothetical imputation model based on *μ*_*Y*, *i*_ instead of *y*_*i*_:

$$ {a}_i={\psi}_0^{\ast }+{\psi}_Y^{\ast }{\mu}_{Y,i}+{\xi}_i, $$

from which it is apparent that ${\psi}_Y^{\ast }=1/{\gamma}_A^{\ast }$. Additionally, across all observations, $\mathrm{Var}\left({\mu}_Y\right)={\gamma_A^{\ast}}^2{\sigma}_A^{\ast 2.}$

In analogy with [16] (referencing [19]) we have that

$$ {\phi}_Y^{\ast }=\frac{\psi_Y^{\ast }{\gamma}_A^{\ast 2}{\sigma}_A^{\ast 2}}{\gamma_A^{\ast 2}{\sigma}_A^{\ast 2}+{\sigma}_Y^{\ast 2}}=\frac{\gamma_A^{\ast }{\sigma}_A^{\ast 2}}{\gamma_A^{\ast 2}{\sigma}_A^{\ast 2}+{\sigma}_Y^{\ast 2}}. $$

Moreover, ${\phi}_0^{\ast }=0$ because *A* and *Y* are both centred.

The imputation model is then used to impute values for the missing *a*_*i*_ s; i.e. for $i\notin \mathcal{J}$, if we knew the true imputation model,

$$ {a}_{i, imp}={\phi}_0^{\ast }+{\phi}_Y^{\ast }{y}_i+{\delta}_i=\frac{\gamma_A^{\ast }{\sigma}_A^{\ast 2}}{\gamma_A^{\ast 2}{\sigma}_A^{\ast 2}+{\sigma}_Y^{\ast 2}}\left({\gamma}_A^{\ast }{a}_i+{\gamma}_U^{\ast }{u}_i+{\epsilon}_i\right)+{\delta}_i. $$

Now consider again the outcome model,

$$ {y}_i={\gamma}_0^{\ast }+{\gamma}_A^{\ast }{a}_i+{\gamma}_U^{\ast }{u}_i+{\epsilon}_i. $$

In the absence of missing data, we would of course simply solve using least squares, and if *γ* = (*γ*_0_, *γ*_*A*_, *γ*_*U*_) and ${\hat{y}}_i\left(\gamma \right)={\gamma}_0+{\gamma}_A{a}_i+{\gamma}_U{u}_i$, then $\overset{\sim }{\gamma }=\mathrm{argmin}\left(\sum \limits_{i=1}^n{\left({y}_i-{\hat{y}}_i\right)}^2\right)$, then of course ${E}_Y\left[{\overset{\sim }{\gamma}}_U\right]={\gamma}_U^{\ast }$.

As we have missing data, rewriting the outcome model to replace the missing *a*_*i*_ s with their imputed versions, for substitution into the least squares formula we have:

$$ {\hat{y}}_i\left(\gamma \right)={\gamma}_0+{\gamma}_A\left(\left(1-{u}_i\right){a}_i+{u}_{\mathrm{i}}{a}_{i, imp}\right)+{\gamma}_U{u}_i. $$

The residual sum of squares can then be written as

$$ \hat{\gamma}=\mathrm{argmin}\sum \limits_{i=1}^n{\left\{\left({\gamma}_0^{\ast }-{\gamma}_0\right)+\left({\gamma}_A^{\ast }-{\gamma}_A\right){a}_i+\left({\gamma}_U^{\ast }-{\gamma}_A{\gamma}_U^{\ast}\kappa -{\gamma}_U\left){u}_i+\right(-{\gamma}_A\kappa {\epsilon}_i-{\gamma}_A{\delta}_i\right){u}_i+\left({\gamma}_A-{\gamma}_A\kappa {\gamma}_A^{\ast}\right){u}_i{a}_i\right\}}^2, $$

where $\kappa =\frac{\gamma_A^{\ast }{\sigma}_A^{\ast 2}}{\gamma_A^{\ast 2}{\sigma}_A^{\ast 2}+{\sigma}_Y^{\ast 2}}$ is a constant.

To consider minimising this expression, consider each bracket in turn. To minimise the first bracket, it is clear that ${E}_Y\left[{\hat{\gamma}}_0\right]={\gamma}_0^{\ast }$. It is also apparent that ${E}_Y\left[{\hat{\gamma}}_A\right]={\gamma}_A^{\ast }$, since we must minimise the second bracket, and the fourth and fifth brackets are additional error contributed by the imputed data, which cannot be reduced. This leaves the third bracket, which is minimised at

$$ {E}_Y\left[{\gamma}_U^{\ast }-{\hat{\gamma}}_A{\gamma}_U^{\ast}\kappa -{\hat{\gamma}}_U\right]=0. $$

Rearranging yields,

$$ {E}_Y\left[{\hat{\gamma}}_U\right]={\gamma}_U^{\ast}\frac{\sigma_Y^{\ast 2}}{\gamma_A^{\ast 2}{\sigma}_A^{\ast 2}+{\sigma}_Y^{\ast 2}}, $$

as claimed.

## Abbreviations

MAR: Missing at random
MCAR: Missing completely at random
MI: Multiple imputation
MIMI: Multiple imputation with missing indicator
MNAR: Missing not at random
