# Supplementary material for: Multiple imputation with missing indicators as proxies for unmeasured variables: simulation study
Source: BMC Med Res Methodol. 2020 Jul 8;20:185. doi: 10.1186/s12874-020-01068-x (PMC7346454; doi:10.1186/s12874-020-01068-x)
Supplement: Supplementary file 1 — Additional file 1. [file 12874_2020_1068_MOESM1_ESM.docx]

**Multiple Imputation with Missing Indicators as Proxies for Unmeasured Variables: Simulation Study**

***Supplemental Material***

Matthew Sperrin and Glen P. Martin


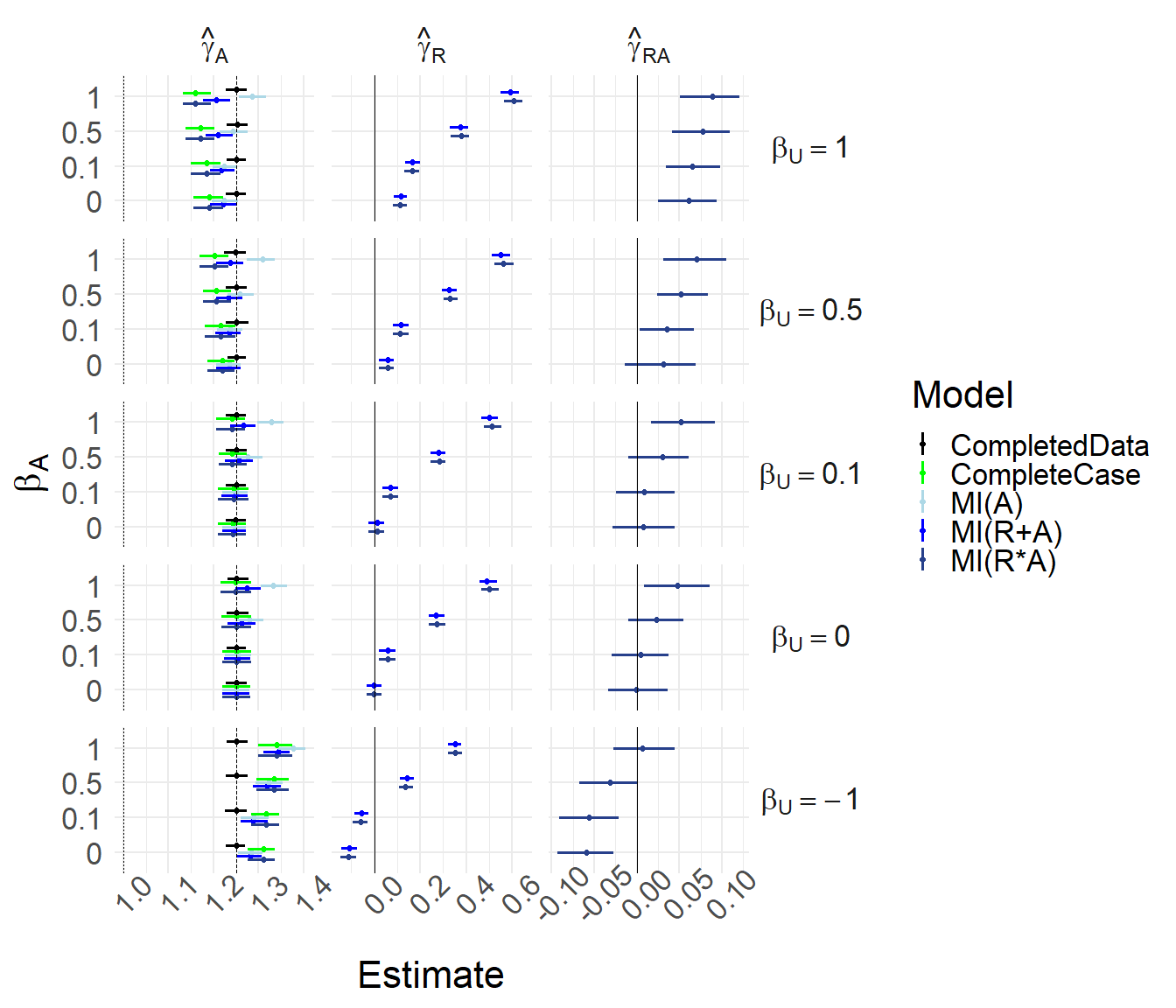


*Figure S1: Results for scenarios (iv) and (v), with $\gamma_{UA}=0.5$. Mean of estimated coefficients across simulations; error bars are 2.5^th^ the 97.5^th^ percentiles. Columns are different parameter estimates, rows are different values of $\beta_{U}$. Within each graph, the y-axis varies $\beta_{A}$.*


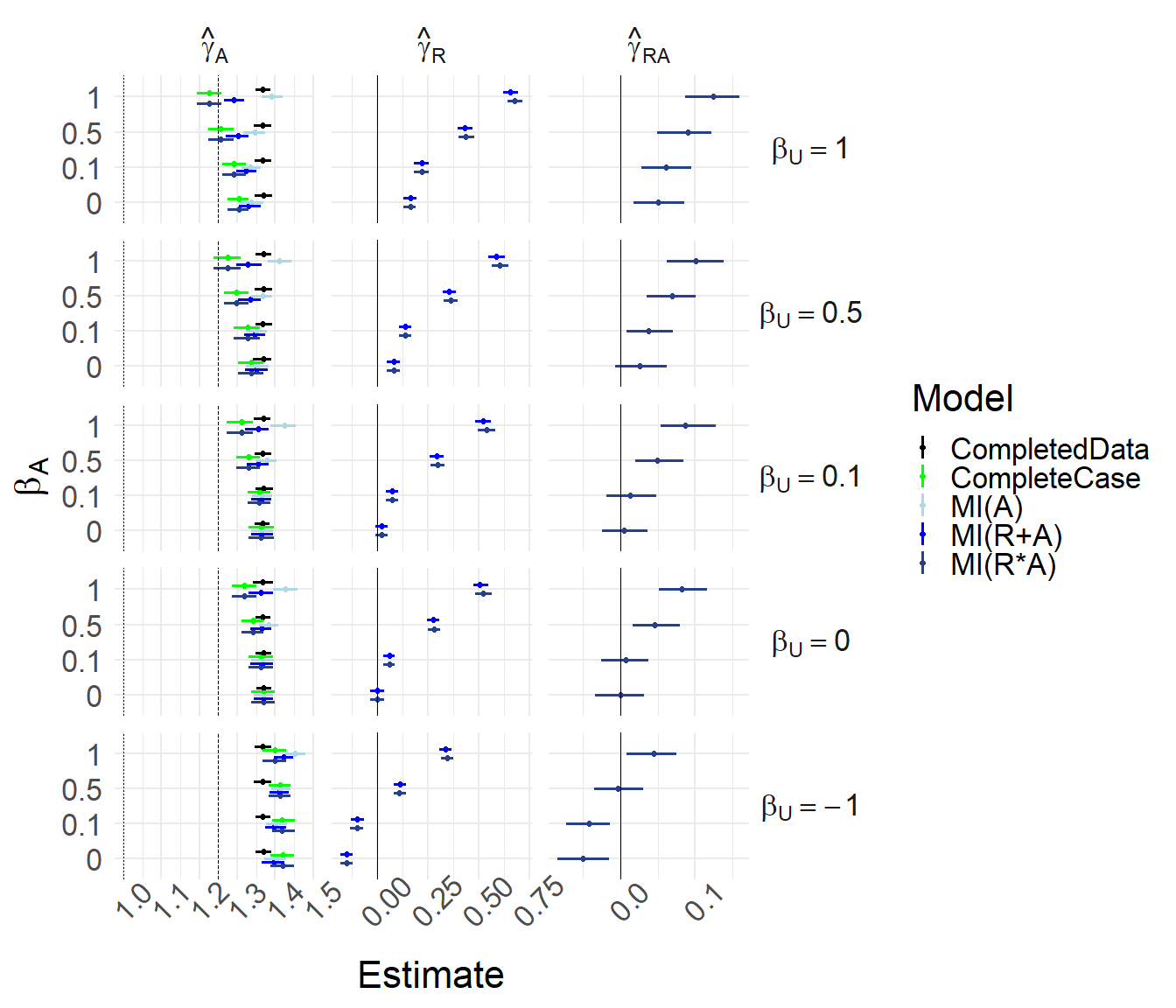


*Figure S2: Results for scenario (vi), with $\gamma_{UA}=0.5$. Mean of estimated coefficients across simulations; error bars are 2.5^th^ the 97.5^th^ percentiles. Columns are different parameter estimates, rows are different values of $\beta_{U}$. Within each graph, the y-axis varies $\beta_{A}$.*


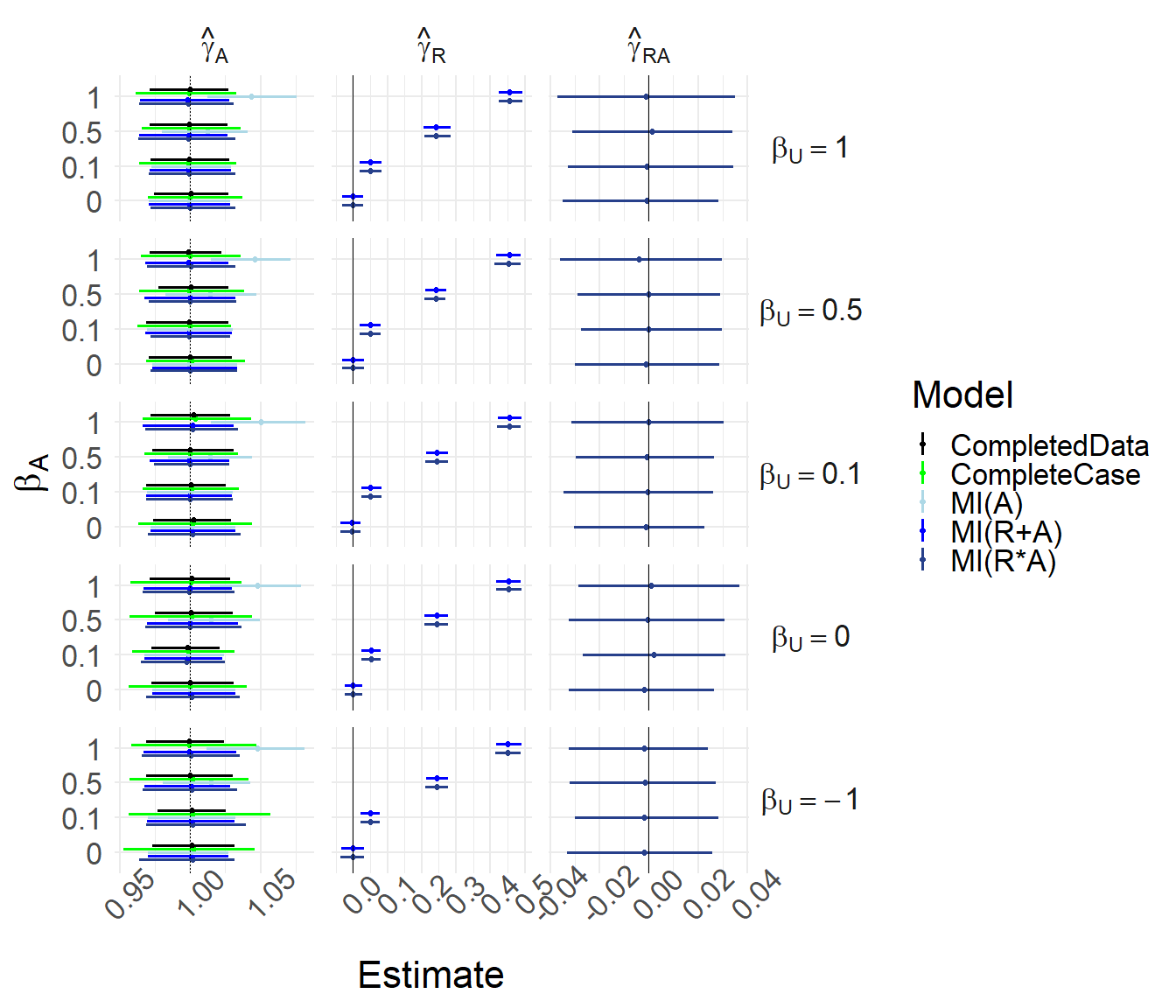


*Figure S3: Results for scenario (vi-U), with $\gamma_{UA}=0$. Mean of estimated coefficients across simulations; error bars are 2.5^th^ the 97.5^th^ percentiles. Columns are different parameter estimates, rows are different values of $\beta_{U}$. Within each graph, the y-axis varies $\beta_{A}$.*

**Supplementary Tables**

*Table S1 (relates to Figure 2):* *Results for scenarios (i)-(iii), with $\gamma_{UA}=0$. Mean estimate of $\gamma_{A}$, width of confidence interval and coverage, for each simulation configuration (rows) and each missing data method (columns).*

|  | **Completed Data** | | |  | **Complete Case** | | | **MI(A)** | | | **MI(R+A)** | | | **MI(R*A)** | | |
| --- | --- | --- | --- | --- | --- | --- | --- | --- | --- | --- | --- | --- | --- | --- | --- | --- |
| **Simulation Parameters** | **Est** | **Width** | **Cov** |  | **Est** | **Width** | **Cov** | **Est** | **Width** | **Cov** | **Est** | **Width** | **Cov** | **Est** | **Width** | **Cov** |
| $\alpha_{U}=$ 1, $\beta_{U}=$ 1 | 1.200 | 0.038 | 0.000 |  | 1.191 | 0.054 | 0.000 | 1.200 | 0.049 | 0.000 | 1.190 | 0.049 | 0.000 | 1.191 | 0.054 | 0.000 |
| $\alpha_{U}=$ 1, $\beta_{U}=$ 0.5 | 1.199 | 0.038 | 0.000 |  | 1.197 | 0.054 | 0.000 | 1.199 | 0.049 | 0.000 | 1.196 | 0.049 | 0.000 | 1.197 | 0.054 | 0.000 |
| $\alpha_{U}=$ 1, $\beta_{U}=$ 0.1 | 1.199 | 0.038 | 0.000 |  | 1.199 | 0.054 | 0.000 | 1.199 | 0.049 | 0.000 | 1.199 | 0.049 | 0.000 | 1.199 | 0.054 | 0.000 |
| $\alpha_{U}=$ 1, $\beta_{U}=$ 0 | 1.201 | 0.038 | 0.000 |  | 1.200 | 0.054 | 0.000 | 1.201 | 0.050 | 0.000 | 1.201 | 0.050 | 0.000 | 1.200 | 0.054 | 0.000 |
| $\alpha_{U}=$ 1, $\beta_{U}=$ -1 | 1.199 | 0.038 | 0.000 |  | 1.189 | 0.054 | 0.000 | 1.198 | 0.049 | 0.000 | 1.189 | 0.049 | 0.000 | 1.189 | 0.054 | 0.000 |
| $\alpha_{U}=$ 1, $R_{A}=1-U$ | 1.200 | 0.038 | 0.000 |  | 1.001 | 0.056 | 0.945 | 1.201 | 0.049 | 0.000 | 1.000 | 0.049 | 0.950 | 1.001 | 0.056 | 0.940 |
| $\alpha_{U}=$ 0.5, $\beta_{U}=$ 1 | 1.117 | 0.042 | 0.000 |  | 1.110 | 0.060 | 0.000 | 1.116 | 0.053 | 0.000 | 1.109 | 0.053 | 0.000 | 1.110 | 0.060 | 0.000 |
| $\alpha_{U}=$ 0.5, $\beta_{U}=$ 0.5 | 1.118 | 0.042 | 0.000 |  | 1.116 | 0.060 | 0.000 | 1.118 | 0.053 | 0.000 | 1.117 | 0.053 | 0.000 | 1.116 | 0.060 | 0.000 |
| $\alpha_{U}=$ 0.5, $\beta_{U}=$ 0.1 | 1.118 | 0.042 | 0.000 |  | 1.118 | 0.060 | 0.000 | 1.118 | 0.054 | 0.000 | 1.117 | 0.054 | 0.000 | 1.118 | 0.060 | 0.000 |
| $\alpha_{U}=$ 0.5, $\beta_{U}=$ 0 | 1.117 | 0.042 | 0.000 |  | 1.118 | 0.060 | 0.000 | 1.117 | 0.052 | 0.000 | 1.117 | 0.052 | 0.000 | 1.118 | 0.060 | 0.000 |
| $\alpha_{U}=$ 0.5, $\beta_{U}=$ -1 | 1.118 | 0.042 | 0.000 |  | 1.112 | 0.060 | 0.000 | 1.119 | 0.054 | 0.000 | 1.112 | 0.054 | 0.000 | 1.112 | 0.060 | 0.000 |
| $\alpha_{U}=$ 0.5, $R_{A}=1-U$ | 1.117 | 0.042 | 0.000 |  | 1.001 | 0.056 | 0.970 | 1.124 | 0.049 | 0.000 | 1.001 | 0.049 | 0.965 | 1.001 | 0.056 | 0.970 |
| $\alpha_{U}=$ 0.1, $\beta_{U}=$ 1 | 1.024 | 0.044 | 0.405 |  | 1.023 | 0.062 | 0.670 | 1.027 | 0.055 | 0.495 | 1.022 | 0.055 | 0.630 | 1.023 | 0.062 | 0.670 |
| $\alpha_{U}=$ 0.1, $\beta_{U}=$ 0.5 | 1.025 | 0.044 | 0.370 |  | 1.024 | 0.062 | 0.695 | 1.025 | 0.055 | 0.595 | 1.024 | 0.055 | 0.635 | 1.024 | 0.062 | 0.690 |
| $\alpha_{U}=$ 0.1, $\beta_{U}=$ 0.1 | 1.025 | 0.044 | 0.370 |  | 1.025 | 0.062 | 0.640 | 1.025 | 0.054 | 0.585 | 1.024 | 0.054 | 0.580 | 1.025 | 0.062 | 0.640 |
| $\alpha_{U}=$ 0.1, $\beta_{U}=$ 0 | 1.026 | 0.044 | 0.385 |  | 1.027 | 0.062 | 0.595 | 1.027 | 0.055 | 0.505 | 1.027 | 0.055 | 0.505 | 1.027 | 0.062 | 0.590 |
| $\alpha_{U}=$ 0.1, $\beta_{U}=$ -1 | 1.025 | 0.044 | 0.420 |  | 1.024 | 0.062 | 0.695 | 1.028 | 0.054 | 0.505 | 1.024 | 0.054 | 0.630 | 1.024 | 0.062 | 0.695 |
| $\alpha_{U}=$ 0.1, $R_{A}=1-U$ | 1.025 | 0.044 | 0.400 |  | 1.000 | 0.055 | 0.970 | 1.070 | 0.049 | 0.000 | 1.000 | 0.049 | 0.940 | 1.000 | 0.055 | 0.965 |
| $\alpha_{U}=$ 0, $\beta_{U}=$ 1 | 1.000 | 0.044 | 0.945 |  | 1.002 | 0.062 | 0.935 | 1.005 | 0.055 | 0.935 | 1.001 | 0.055 | 0.955 | 1.002 | 0.062 | 0.935 |
| $\alpha_{U}=$ 0, $\beta_{U}=$ 0.5 | 1.001 | 0.044 | 0.940 |  | 1.001 | 0.062 | 0.955 | 1.003 | 0.055 | 0.940 | 1.002 | 0.055 | 0.940 | 1.001 | 0.062 | 0.955 |
| $\alpha_{U}=$ 0, $\beta_{U}=$ 0.1 | 0.999 | 0.044 | 0.940 |  | 0.998 | 0.062 | 0.950 | 0.998 | 0.054 | 0.935 | 0.998 | 0.054 | 0.935 | 0.998 | 0.062 | 0.950 |
| $\alpha_{U}=$ 0, $\beta_{U}=$ 0 | 1.000 | 0.044 | 0.945 |  | 1.000 | 0.062 | 0.955 | 1.000 | 0.056 | 0.945 | 1.000 | 0.056 | 0.945 | 1.000 | 0.062 | 0.955 |
| $\alpha_{U}=$ 0, $\beta_{U}=$ -1 | 1.000 | 0.044 | 0.960 |  | 0.998 | 0.062 | 0.960 | 1.003 | 0.055 | 0.950 | 0.999 | 0.055 | 0.950 | 0.998 | 0.062 | 0.960 |
| $\alpha_{U}=$ 0, $R_{A}=1-U$ | 1.000 | 0.044 | 0.950 |  | 1.000 | 0.055 | 0.930 | 1.058 | 0.049 | 0.000 | 1.000 | 0.049 | 0.965 | 1.000 | 0.055 | 0.930 |

*Table S2 (relates to Figure 3):* *Results for scenarios (i)-(iii), with $\gamma_{UA}=0.5$. Mean estimate of $\gamma_{A}$, width of confidence interval and coverage, for each simulation configuration (rows) and each missing data method (columns).*

|  | **Completed Data** | | | **Complete Case** | | | **MI(A)** | | | **MI(R+A)** | | | **MI(R*A)** | | |
| --- | --- | --- | --- | --- | --- | --- | --- | --- | --- | --- | --- | --- | --- | --- | --- |
| **Simulation Parameters** | **Est** | **Width** | **Cov** | **Est** | **Width** | **Cov** | **Est** | **Width** | **Cov** | **Est** | **Width** | **Cov** | **Est** | **Width** | **Cov** |
| $\alpha_{U}=$ 1, $\beta_{U}=$ 1 | 1.451 | 0.039 | 0 | 1.392 | 0.056 | 0.000 | 1.421 | 0.051 | 0 | 1.411 | 0.051 | 0 | 1.392 | 0.057 | 0.000 |
| $\alpha_{U}=$ 1, $\beta_{U}=$ 0.5 | 1.450 | 0.039 | 0 | 1.422 | 0.056 | 0.000 | 1.434 | 0.051 | 0 | 1.432 | 0.051 | 0 | 1.422 | 0.056 | 0.000 |
| $\alpha_{U}=$ 1, $\beta_{U}=$ 0.1 | 1.451 | 0.039 | 0 | 1.445 | 0.056 | 0.000 | 1.447 | 0.051 | 0 | 1.447 | 0.051 | 0 | 1.445 | 0.056 | 0.000 |
| $\alpha_{U}=$ 1, $\beta_{U}=$ 0 | 1.450 | 0.039 | 0 | 1.450 | 0.056 | 0.000 | 1.450 | 0.050 | 0 | 1.450 | 0.050 | 0 | 1.450 | 0.056 | 0.000 |
| $\alpha_{U}=$ 1, $\beta_{U}=$ -1 | 1.450 | 0.039 | 0 | 1.490 | 0.055 | 0.000 | 1.481 | 0.050 | 0 | 1.472 | 0.050 | 0 | 1.490 | 0.055 | 0.000 |
| $\alpha_{U}=$ 1, $R_{A}=1-U$ | 1.450 | 0.039 | 0 | 1.000 | 0.055 | 0.925 | 1.321 | 0.051 | 0 | 1.135 | 0.049 | 0 | 1.000 | 0.059 | 0.950 |
| $\alpha_{U}=$ 0.5, $\beta_{U}=$ 1 | 1.368 | 0.043 | 0 | 1.304 | 0.061 | 0.000 | 1.337 | 0.055 | 0 | 1.330 | 0.055 | 0 | 1.304 | 0.062 | 0.000 |
| $\alpha_{U}=$ 0.5, $\beta_{U}=$ 0.5 | 1.368 | 0.043 | 0 | 1.337 | 0.061 | 0.000 | 1.352 | 0.055 | 0 | 1.350 | 0.055 | 0 | 1.337 | 0.062 | 0.000 |
| $\alpha_{U}=$ 0.5, $\beta_{U}=$ 0.1 | 1.367 | 0.043 | 0 | 1.361 | 0.061 | 0.000 | 1.364 | 0.055 | 0 | 1.364 | 0.055 | 0 | 1.361 | 0.061 | 0.000 |
| $\alpha_{U}=$ 0.5, $\beta_{U}=$ 0 | 1.369 | 0.043 | 0 | 1.369 | 0.061 | 0.000 | 1.368 | 0.054 | 0 | 1.368 | 0.054 | 0 | 1.369 | 0.061 | 0.000 |
| $\alpha_{U}=$ 0.5, $\beta_{U}=$ -1 | 1.369 | 0.043 | 0 | 1.421 | 0.061 | 0.000 | 1.403 | 0.054 | 0 | 1.396 | 0.054 | 0 | 1.421 | 0.060 | 0.000 |
| $\alpha_{U}=$ 0.5, $R_{A}=1-U$ | 1.368 | 0.043 | 0 | 1.001 | 0.055 | 0.935 | 1.244 | 0.050 | 0 | 1.136 | 0.049 | 0 | 1.001 | 0.059 | 0.950 |
| $\alpha_{U}=$ 0.1, $\beta_{U}=$ 1 | 1.275 | 0.045 | 0 | 1.212 | 0.063 | 0.000 | 1.246 | 0.056 | 0 | 1.243 | 0.056 | 0 | 1.212 | 0.064 | 0.000 |
| $\alpha_{U}=$ 0.1, $\beta_{U}=$ 0.5 | 1.276 | 0.045 | 0 | 1.244 | 0.063 | 0.000 | 1.260 | 0.057 | 0 | 1.259 | 0.057 | 0 | 1.244 | 0.064 | 0.000 |
| $\alpha_{U}=$ 0.1, $\beta_{U}=$ 0.1 | 1.275 | 0.045 | 0 | 1.269 | 0.063 | 0.000 | 1.271 | 0.056 | 0 | 1.271 | 0.056 | 0 | 1.269 | 0.063 | 0.000 |
| $\alpha_{U}=$ 0.1, $\beta_{U}=$ 0 | 1.275 | 0.045 | 0 | 1.275 | 0.063 | 0.000 | 1.274 | 0.056 | 0 | 1.274 | 0.056 | 0 | 1.275 | 0.063 | 0.000 |
| $\alpha_{U}=$ 0.1, $\beta_{U}=$ -1 | 1.275 | 0.045 | 0 | 1.335 | 0.063 | 0.000 | 1.310 | 0.057 | 0 | 1.306 | 0.057 | 0 | 1.335 | 0.062 | 0.000 |
| $\alpha_{U}=$ 0.1, $R_{A}=1-U$ | 1.275 | 0.045 | 0 | 1.000 | 0.055 | 0.950 | 1.191 | 0.050 | 0 | 1.136 | 0.049 | 0 | 1.000 | 0.059 | 0.965 |
| $\alpha_{U}=$ 0, $\beta_{U}=$ 1 | 1.251 | 0.045 | 0 | 1.190 | 0.063 | 0.000 | 1.224 | 0.056 | 0 | 1.221 | 0.056 | 0 | 1.190 | 0.064 | 0.000 |
| $\alpha_{U}=$ 0, $\beta_{U}=$ 0.5 | 1.251 | 0.045 | 0 | 1.219 | 0.063 | 0.000 | 1.235 | 0.056 | 0 | 1.235 | 0.056 | 0 | 1.219 | 0.064 | 0.000 |
| $\alpha_{U}=$ 0, $\beta_{U}=$ 0.1 | 1.249 | 0.045 | 0 | 1.243 | 0.064 | 0.000 | 1.246 | 0.056 | 0 | 1.246 | 0.056 | 0 | 1.243 | 0.064 | 0.000 |
| $\alpha_{U}=$ 0, $\beta_{U}=$ 0 | 1.250 | 0.045 | 0 | 1.250 | 0.063 | 0.000 | 1.250 | 0.056 | 0 | 1.250 | 0.056 | 0 | 1.250 | 0.063 | 0.000 |
| $\alpha_{U}=$ 0, $\beta_{U}=$ -1 | 1.250 | 0.045 | 0 | 1.310 | 0.063 | 0.000 | 1.284 | 0.056 | 0 | 1.281 | 0.056 | 0 | 1.310 | 0.062 | 0.000 |
| $\alpha_{U}=$ 0, $R_{A}=1-U$ | 1.250 | 0.045 | 0 | 1.001 | 0.055 | 0.970 | 1.180 | 0.048 | 0 | 1.136 | 0.049 | 0 | 1.001 | 0.059 | 0.975 |

*Table S3 (relates to Figure 4):* *Results for scenarios (i-U)-(iii-U), with $\gamma_{UA}=0$. Mean estimate of $\gamma_{A}$, width of confidence interval and coverage, for each simulation configuration (rows) and each missing data method (columns).*

|  | **Completed Data** | | | **Complete Case** | | | **MI(A)** | | | **MI(R+A)** | | | **MI(R*A)** | | |
| --- | --- | --- | --- | --- | --- | --- | --- | --- | --- | --- | --- | --- | --- | --- | --- |
| **Simulation Parameters** | **Est** | **Width** | **Cov** | **Est** | **Width** | **Cov** | **Est** | **Width** | **Cov** | **Est** | **Width** | **Cov** | **Est** | **Width** | **Cov** |
| $\alpha_{U}=$ 1, $\beta_{U}=$ 1 | 1.000 | 0.056 | 0.960 | 1.001 | 0.070 | 0.930 | 1.000 | 0.063 | 0.955 | 1.000 | 0.063 | 0.955 | 1.000 | 0.065 | 0.965 |
| $\alpha_{U}=$ 1, $\beta_{U}=$ 0.5 | 1.000 | 0.055 | 0.945 | 1.000 | 0.074 | 0.940 | 0.999 | 0.065 | 0.945 | 0.999 | 0.065 | 0.945 | 1.000 | 0.067 | 0.950 |
| $\alpha_{U}=$ 1, $\beta_{U}=$ 0.1 | 0.999 | 0.055 | 0.945 | 0.998 | 0.077 | 0.955 | 0.999 | 0.067 | 0.970 | 0.999 | 0.067 | 0.970 | 0.999 | 0.070 | 0.970 |
| $\alpha_{U}=$ 1, $\beta_{U}=$ 0 | 1.000 | 0.055 | 0.945 | 0.999 | 0.079 | 0.940 | 1.000 | 0.066 | 0.960 | 1.000 | 0.066 | 0.960 | 0.999 | 0.071 | 0.965 |
| $\alpha_{U}=$ 1, $\beta_{U}=$ -1 | 0.999 | 0.055 | 0.950 | 0.998 | 0.090 | 0.955 | 0.998 | 0.069 | 0.955 | 0.998 | 0.069 | 0.955 | 0.999 | 0.078 | 0.960 |
| $\alpha_{U}=$ 0.5, $\beta_{U}=$ 1 | 1.000 | 0.055 | 0.965 | 1.000 | 0.070 | 0.945 | 0.999 | 0.064 | 0.955 | 0.999 | 0.064 | 0.955 | 1.000 | 0.066 | 0.955 |
| $\alpha_{U}=$ 0.5, $\beta_{U}=$ 0.5 | 1.000 | 0.055 | 0.940 | 1.001 | 0.074 | 0.940 | 0.999 | 0.065 | 0.970 | 0.999 | 0.065 | 0.970 | 1.000 | 0.068 | 0.955 |
| $\alpha_{U}=$ 0.5, $\beta_{U}=$ 0.1 | 1.002 | 0.056 | 0.945 | 1.002 | 0.078 | 0.915 | 1.001 | 0.067 | 0.960 | 1.001 | 0.067 | 0.960 | 1.002 | 0.071 | 0.960 |
| $\alpha_{U}=$ 0.5, $\beta_{U}=$ 0 | 1.000 | 0.055 | 0.945 | 1.000 | 0.078 | 0.940 | 1.000 | 0.066 | 0.965 | 1.000 | 0.066 | 0.965 | 1.001 | 0.071 | 0.955 |
| $\alpha_{U}=$ 0.5, $\beta_{U}=$ -1 | 1.001 | 0.055 | 0.945 | 1.003 | 0.090 | 0.940 | 1.000 | 0.069 | 0.975 | 1.000 | 0.069 | 0.975 | 1.002 | 0.079 | 0.985 |
| $\alpha_{U}=$ 0.1, $\beta_{U}=$ 1 | 1.000 | 0.055 | 0.940 | 1.000 | 0.070 | 0.950 | 1.000 | 0.064 | 0.945 | 1.000 | 0.064 | 0.945 | 1.000 | 0.065 | 0.960 |
| $\alpha_{U}=$ 0.1, $\beta_{U}=$ 0.5 | 1.000 | 0.055 | 0.945 | 0.999 | 0.074 | 0.980 | 0.999 | 0.066 | 0.965 | 0.999 | 0.066 | 0.960 | 0.999 | 0.068 | 0.960 |
| $\alpha_{U}=$ 0.1, $\beta_{U}=$ 0.1 | 1.000 | 0.055 | 0.965 | 1.001 | 0.077 | 0.945 | 0.999 | 0.066 | 0.965 | 0.999 | 0.066 | 0.970 | 1.000 | 0.070 | 0.975 |
| $\alpha_{U}=$ 0.1, $\beta_{U}=$ 0 | 1.001 | 0.055 | 0.940 | 1.003 | 0.078 | 0.960 | 1.001 | 0.067 | 0.970 | 1.001 | 0.067 | 0.970 | 1.002 | 0.072 | 0.970 |
| $\alpha_{U}=$ 0.1, $\beta_{U}=$ -1 | 0.999 | 0.055 | 0.945 | 0.998 | 0.090 | 0.965 | 0.999 | 0.071 | 0.980 | 0.999 | 0.071 | 0.980 | 0.999 | 0.080 | 0.975 |
| $\alpha_{U}=$ 0, $\beta_{U}=$ 1 | 1.001 | 0.055 | 0.950 | 1.002 | 0.070 | 0.925 | 1.001 | 0.063 | 0.945 | 1.001 | 0.063 | 0.945 | 1.002 | 0.065 | 0.955 |
| $\alpha_{U}=$ 0, $\beta_{U}=$ 0.5 | 1.001 | 0.055 | 0.940 | 1.000 | 0.074 | 0.950 | 1.001 | 0.064 | 0.965 | 1.001 | 0.064 | 0.965 | 1.001 | 0.068 | 0.970 |
| $\alpha_{U}=$ 0, $\beta_{U}=$ 0.1 | 1.000 | 0.055 | 0.940 | 0.999 | 0.077 | 0.945 | 0.998 | 0.065 | 0.970 | 0.998 | 0.065 | 0.970 | 0.999 | 0.070 | 0.960 |
| $\alpha_{U}=$ 0, $\beta_{U}=$ 0 | 1.001 | 0.056 | 0.960 | 1.002 | 0.078 | 0.955 | 1.001 | 0.066 | 0.975 | 1.001 | 0.067 | 0.975 | 1.001 | 0.071 | 0.965 |
| $\alpha_{U}=$ 0, $\beta_{U}=$ -1 | 1.000 | 0.055 | 0.925 | 0.999 | 0.090 | 0.940 | 1.000 | 0.070 | 0.955 | 1.000 | 0.070 | 0.955 | 0.999 | 0.079 | 0.975 |

*Table S4 (relates to Figure 5):* *Results for scenarios (iv) and (v), with $\gamma_{UA}=0$. Mean estimate of $\gamma_{A}$, width of confidence interval and coverage, for each simulation configuration (rows) and each missing data method (columns).*

|  | **Completed Data** | | | **Complete Case** | | | **MI(A)** | | | **MI(R+A)** | | | **MI(R*A)** | | |
| --- | --- | --- | --- | --- | --- | --- | --- | --- | --- | --- | --- | --- | --- | --- | --- |
| **Simulation Parameters** | **Est** | **Width** | **Cov** | **Est** | **Width** | **Cov** | **Est** | **Width** | **Cov** | **Est** | **Width** | **Cov** | **Est** | **Width** | **Cov** |
| $\beta_{U}=$ 1, $\beta_{A}=$ 0 | 1.000 | 0.044 | 0.945 | 1.002 | 0.062 | 0.935 | 1.005 | 0.055 | 0.935 | 1.001 | 0.055 | 0.955 | 1.002 | 0.062 | 0.935 |
| $\beta_{U}=$ 1, $\beta_{A}=$ 0.1 | 1.001 | 0.044 | 0.965 | 0.995 | 0.062 | 0.965 | 1.002 | 0.054 | 0.945 | 0.994 | 0.054 | 0.940 | 0.995 | 0.062 | 0.965 |
| $\beta_{U}=$ 1, $\beta_{A}=$ 0.5 | 1.001 | 0.044 | 0.970 | 0.973 | 0.063 | 0.620 | 1.003 | 0.057 | 0.940 | 0.973 | 0.056 | 0.535 | 0.973 | 0.063 | 0.620 |
| $\beta_{U}=$ 1, $\beta_{A}=$ 1 | 1.000 | 0.044 | 0.965 | 0.954 | 0.067 | 0.260 | 1.023 | 0.061 | 0.675 | 0.954 | 0.060 | 0.180 | 0.954 | 0.067 | 0.260 |
| $\beta_{U}=$ 0.5, $\beta_{A}=$ 0 | 1.001 | 0.044 | 0.940 | 1.001 | 0.062 | 0.955 | 1.003 | 0.055 | 0.940 | 1.002 | 0.055 | 0.940 | 1.001 | 0.062 | 0.955 |
| $\beta_{U}=$ 0.5, $\beta_{A}=$ 0.1 | 1.000 | 0.044 | 0.950 | 0.997 | 0.062 | 0.955 | 0.999 | 0.055 | 0.970 | 0.996 | 0.055 | 0.965 | 0.997 | 0.062 | 0.955 |
| $\beta_{U}=$ 0.5, $\beta_{A}=$ 0.5 | 0.998 | 0.044 | 0.930 | 0.984 | 0.064 | 0.810 | 1.005 | 0.058 | 0.935 | 0.984 | 0.057 | 0.790 | 0.984 | 0.064 | 0.810 |
| $\beta_{U}=$ 0.5, $\beta_{A}=$ 1 | 1.002 | 0.044 | 0.940 | 0.976 | 0.068 | 0.695 | 1.036 | 0.061 | 0.380 | 0.976 | 0.061 | 0.635 | 0.976 | 0.068 | 0.690 |
| $\beta_{U}=$ 0.1, $\beta_{A}=$ 0 | 0.999 | 0.044 | 0.940 | 0.998 | 0.062 | 0.950 | 0.998 | 0.054 | 0.935 | 0.998 | 0.054 | 0.935 | 0.998 | 0.062 | 0.950 |
| $\beta_{U}=$ 0.1, $\beta_{A}=$ 0.1 | 1.001 | 0.044 | 0.960 | 1.002 | 0.062 | 0.965 | 1.002 | 0.055 | 0.930 | 1.001 | 0.055 | 0.935 | 1.002 | 0.062 | 0.960 |
| $\beta_{U}=$ 0.1, $\beta_{A}=$ 0.5 | 1.000 | 0.044 | 0.955 | 0.998 | 0.064 | 0.925 | 1.012 | 0.057 | 0.845 | 0.997 | 0.057 | 0.940 | 0.998 | 0.064 | 0.925 |
| $\beta_{U}=$ 0.1, $\beta_{A}=$ 1 | 1.001 | 0.044 | 0.955 | 0.995 | 0.068 | 0.945 | 1.047 | 0.062 | 0.160 | 0.997 | 0.061 | 0.940 | 0.995 | 0.068 | 0.945 |
| $\beta_{U}=$ 0, $\beta_{A}=$ 0 | 1.000 | 0.044 | 0.945 | 1.000 | 0.062 | 0.955 | 1.000 | 0.056 | 0.945 | 1.000 | 0.056 | 0.945 | 1.000 | 0.062 | 0.955 |
| $\beta_{U}=$ 0, $\beta_{A}=$ 0.1 | 1.001 | 0.044 | 0.945 | 1.000 | 0.062 | 0.945 | 1.001 | 0.055 | 0.955 | 1.000 | 0.055 | 0.950 | 1.000 | 0.062 | 0.945 |
| $\beta_{U}=$ 0, $\beta_{A}=$ 0.5 | 0.999 | 0.044 | 0.930 | 0.999 | 0.064 | 0.960 | 1.013 | 0.055 | 0.845 | 0.999 | 0.055 | 0.935 | 0.999 | 0.064 | 0.960 |
| $\beta_{U}=$ 0, $\beta_{A}=$ 1 | 1.001 | 0.044 | 0.950 | 1.001 | 0.068 | 0.960 | 1.048 | 0.062 | 0.110 | 1.000 | 0.061 | 0.945 | 1.001 | 0.068 | 0.960 |
| $\beta_{U}=$ -1, $\beta_{A}=$ 0 | 1.000 | 0.044 | 0.960 | 0.998 | 0.062 | 0.960 | 1.003 | 0.055 | 0.950 | 0.999 | 0.055 | 0.950 | 0.998 | 0.062 | 0.960 |
| $\beta_{U}=$ -1, $\beta_{A}=$ 0.1 | 1.000 | 0.044 | 0.930 | 1.006 | 0.062 | 0.920 | 1.007 | 0.055 | 0.925 | 1.006 | 0.055 | 0.925 | 1.006 | 0.062 | 0.925 |
| $\beta_{U}=$ -1, $\beta_{A}=$ 0.5 | 1.000 | 0.044 | 0.955 | 1.028 | 0.063 | 0.610 | 1.031 | 0.056 | 0.425 | 1.028 | 0.056 | 0.515 | 1.028 | 0.063 | 0.610 |
| $\beta_{U}=$ -1, $\beta_{A}=$ 1 | 1.000 | 0.044 | 0.935 | 1.047 | 0.067 | 0.245 | 1.071 | 0.060 | 0.010 | 1.046 | 0.060 | 0.190 | 1.047 | 0.067 | 0.250 |

*Table S5 (relates to Figure 6):* *Results for scenarios (iv-U) and (v-U), with $\gamma_{UA}=0$. Mean estimate of $\gamma_{A}$, width of confidence interval and coverage, for each simulation configuration (rows) and each missing data method (columns).*

|  | **Completed Data** | | | **Complete Case** | | | **MI(A)** | | | **MI(R+A)** | | | **MI(R*A)** | | |
| --- | --- | --- | --- | --- | --- | --- | --- | --- | --- | --- | --- | --- | --- | --- | --- |
| **Simulation Parameters** | **Est** | **Width** | **Cov** | **Est** | **Width** | **Cov** | **Est** | **Width** | **Cov** | **Est** | **Width** | **Cov** | **Est** | **Width** | **Cov** |
| $\beta_{U}=$ 1, $\beta_{A}=$ 0 | 1.001 | 0.055 | 0.950 | 1.002 | 0.070 | 0.925 | 1.001 | 0.063 | 0.945 | 1.001 | 0.063 | 0.945 | 1.002 | 0.065 | 0.955 |
| $\beta_{U}=$ 1, $\beta_{A}=$ 0.1 | 1.000 | 0.055 | 0.965 | 1.000 | 0.070 | 0.960 | 1.000 | 0.063 | 0.970 | 1.000 | 0.063 | 0.970 | 1.001 | 0.066 | 0.980 |
| $\beta_{U}=$ 1, $\beta_{A}=$ 0.5 | 1.002 | 0.055 | 0.945 | 1.002 | 0.073 | 0.945 | 1.016 | 0.065 | 0.860 | 1.002 | 0.065 | 0.950 | 1.002 | 0.067 | 0.965 |
| $\beta_{U}=$ 1, $\beta_{A}=$ 1 | 0.999 | 0.056 | 0.955 | 1.001 | 0.078 | 0.950 | 1.045 | 0.070 | 0.280 | 0.999 | 0.070 | 0.970 | 1.000 | 0.072 | 0.970 |
| $\beta_{U}=$ 0.5, $\beta_{A}=$ 0 | 1.001 | 0.055 | 0.940 | 1.000 | 0.074 | 0.950 | 1.001 | 0.064 | 0.965 | 1.001 | 0.064 | 0.965 | 1.001 | 0.068 | 0.970 |
| $\beta_{U}=$ 0.5, $\beta_{A}=$ 0.1 | 1.001 | 0.055 | 0.950 | 1.001 | 0.074 | 0.945 | 1.001 | 0.065 | 0.955 | 1.000 | 0.065 | 0.955 | 1.001 | 0.068 | 0.950 |
| $\beta_{U}=$ 0.5, $\beta_{A}=$ 0.5 | 0.999 | 0.056 | 0.965 | 1.001 | 0.076 | 0.940 | 1.013 | 0.066 | 0.895 | 0.999 | 0.066 | 0.970 | 0.999 | 0.070 | 0.960 |
| $\beta_{U}=$ 0.5, $\beta_{A}=$ 1 | 1.002 | 0.055 | 0.930 | 1.002 | 0.082 | 0.940 | 1.050 | 0.072 | 0.220 | 1.002 | 0.071 | 0.955 | 1.002 | 0.075 | 0.950 |
| $\beta_{U}=$ 0.1, $\beta_{A}=$ 0 | 1.000 | 0.055 | 0.940 | 0.999 | 0.077 | 0.945 | 0.998 | 0.065 | 0.970 | 0.998 | 0.065 | 0.970 | 0.999 | 0.070 | 0.960 |
| $\beta_{U}=$ 0.1, $\beta_{A}=$ 0.1 | 1.003 | 0.055 | 0.945 | 1.003 | 0.078 | 0.940 | 1.002 | 0.066 | 0.950 | 1.002 | 0.066 | 0.950 | 1.003 | 0.070 | 0.965 |
| $\beta_{U}=$ 0.1, $\beta_{A}=$ 0.5 | 1.001 | 0.056 | 0.940 | 1.003 | 0.080 | 0.945 | 1.015 | 0.068 | 0.835 | 1.001 | 0.068 | 0.960 | 1.002 | 0.073 | 0.950 |
| $\beta_{U}=$ 0.1, $\beta_{A}=$ 1 | 1.001 | 0.056 | 0.935 | 0.999 | 0.085 | 0.970 | 1.048 | 0.073 | 0.240 | 1.000 | 0.073 | 0.975 | 0.999 | 0.078 | 0.990 |
| $\beta_{U}=$ 0, $\beta_{A}=$ 0 | 1.001 | 0.056 | 0.960 | 1.002 | 0.078 | 0.955 | 1.001 | 0.066 | 0.975 | 1.001 | 0.067 | 0.975 | 1.001 | 0.071 | 0.965 |
| $\beta_{U}=$ 0, $\beta_{A}=$ 0.1 | 1.001 | 0.056 | 0.925 | 0.999 | 0.079 | 0.935 | 1.001 | 0.066 | 0.960 | 1.000 | 0.066 | 0.955 | 0.999 | 0.071 | 0.935 |
| $\beta_{U}=$ 0, $\beta_{A}=$ 0.5 | 0.999 | 0.055 | 0.935 | 0.998 | 0.081 | 0.935 | 1.013 | 0.068 | 0.880 | 0.998 | 0.068 | 0.945 | 0.999 | 0.073 | 0.960 |
| $\beta_{U}=$ 0, $\beta_{A}=$ 1 | 1.000 | 0.055 | 0.980 | 1.000 | 0.086 | 0.985 | 1.048 | 0.073 | 0.260 | 0.999 | 0.073 | 0.970 | 0.999 | 0.078 | 0.985 |
| $\beta_{U}=$ -1, $\beta_{A}=$ 0 | 1.000 | 0.055 | 0.925 | 0.999 | 0.090 | 0.940 | 1.000 | 0.070 | 0.955 | 1.000 | 0.070 | 0.955 | 0.999 | 0.079 | 0.975 |
| $\beta_{U}=$ -1, $\beta_{A}=$ 0.1 | 1.000 | 0.056 | 0.955 | 1.002 | 0.090 | 0.950 | 1.002 | 0.069 | 0.960 | 1.001 | 0.069 | 0.965 | 1.001 | 0.080 | 0.970 |
| $\beta_{U}=$ -1, $\beta_{A}=$ 0.5 | 0.999 | 0.055 | 0.960 | 1.000 | 0.092 | 0.935 | 1.013 | 0.071 | 0.900 | 0.999 | 0.071 | 0.965 | 1.000 | 0.081 | 0.945 |
| $\beta_{U}=$ -1, $\beta_{A}=$ 1 | 1.000 | 0.056 | 0.965 | 1.001 | 0.097 | 0.970 | 1.048 | 0.076 | 0.315 | 1.001 | 0.075 | 0.975 | 1.002 | 0.084 | 0.990 |

*Table S6 (relates to Figure 7):* *Results for scenario (vi), with $\gamma_{UA}=0$ and $\alpha_{U}=0.5$. Mean estimate of $\gamma_{A}$, width of confidence interval and coverage, for each simulation configuration (rows) and each missing data method (columns).*

|  | **Completed Data** | | | **Complete Case** | | | **MI(A)** | | | **MI(R+A)** | | | **MI(R*A)** | | |
| --- | --- | --- | --- | --- | --- | --- | --- | --- | --- | --- | --- | --- | --- | --- | --- |
| **Simulation Parameters** | **Est** | **Width** | **Cov** | **Est** | **Width** | **Cov** | **Est** | **Width** | **Cov** | **Est** | **Width** | **Cov** | **Est** | **Width** | **Cov** |
| $\beta_{U}=$ 1, $\beta_{A}=$ 0 | 1.117 | 0.042 | 0 | 1.110 | 0.060 | 0.000 | 1.116 | 0.053 | 0 | 1.109 | 0.053 | 0.000 | 1.110 | 0.060 | 0.00 |
| $\beta_{U}=$ 1, $\beta_{A}=$ 0.1 | 1.118 | 0.042 | 0 | 1.105 | 0.060 | 0.000 | 1.117 | 0.053 | 0 | 1.105 | 0.053 | 0.000 | 1.105 | 0.060 | 0.00 |
| $\beta_{U}=$ 1, $\beta_{A}=$ 0.5 | 1.117 | 0.042 | 0 | 1.084 | 0.062 | 0.000 | 1.127 | 0.055 | 0 | 1.085 | 0.055 | 0.000 | 1.084 | 0.062 | 0.00 |
| $\beta_{U}=$ 1, $\beta_{A}=$ 1 | 1.118 | 0.042 | 0 | 1.065 | 0.066 | 0.025 | 1.158 | 0.059 | 0 | 1.065 | 0.059 | 0.005 | 1.065 | 0.066 | 0.03 |
| $\beta_{U}=$ 0.5, $\beta_{A}=$ 0 | 1.118 | 0.042 | 0 | 1.116 | 0.060 | 0.000 | 1.118 | 0.053 | 0 | 1.117 | 0.053 | 0.000 | 1.116 | 0.060 | 0.00 |
| $\beta_{U}=$ 0.5, $\beta_{A}=$ 0.1 | 1.116 | 0.042 | 0 | 1.110 | 0.060 | 0.000 | 1.116 | 0.053 | 0 | 1.111 | 0.053 | 0.000 | 1.110 | 0.060 | 0.00 |
| $\beta_{U}=$ 0.5, $\beta_{A}=$ 0.5 | 1.118 | 0.042 | 0 | 1.102 | 0.062 | 0.000 | 1.130 | 0.055 | 0 | 1.102 | 0.055 | 0.000 | 1.102 | 0.062 | 0.00 |
| $\beta_{U}=$ 0.5, $\beta_{A}=$ 1 | 1.117 | 0.042 | 0 | 1.093 | 0.066 | 0.000 | 1.168 | 0.059 | 0 | 1.092 | 0.059 | 0.000 | 1.093 | 0.066 | 0.00 |
| $\beta_{U}=$ 0.1, $\beta_{A}=$ 0 | 1.118 | 0.042 | 0 | 1.118 | 0.060 | 0.000 | 1.118 | 0.054 | 0 | 1.117 | 0.054 | 0.000 | 1.118 | 0.060 | 0.00 |
| $\beta_{U}=$ 0.1, $\beta_{A}=$ 0.1 | 1.118 | 0.042 | 0 | 1.117 | 0.060 | 0.000 | 1.118 | 0.052 | 0 | 1.117 | 0.052 | 0.000 | 1.117 | 0.060 | 0.00 |
| $\beta_{U}=$ 0.1, $\beta_{A}=$ 0.5 | 1.118 | 0.042 | 0 | 1.114 | 0.062 | 0.000 | 1.133 | 0.054 | 0 | 1.114 | 0.054 | 0.000 | 1.114 | 0.062 | 0.00 |
| $\beta_{U}=$ 0.1, $\beta_{A}=$ 1 | 1.119 | 0.042 | 0 | 1.115 | 0.066 | 0.000 | 1.175 | 0.059 | 0 | 1.114 | 0.059 | 0.000 | 1.115 | 0.066 | 0.00 |
| $\beta_{U}=$ 0, $\beta_{A}=$ 0 | 1.117 | 0.042 | 0 | 1.118 | 0.060 | 0.000 | 1.117 | 0.052 | 0 | 1.117 | 0.052 | 0.000 | 1.118 | 0.060 | 0.00 |
| $\beta_{U}=$ 0, $\beta_{A}=$ 0.1 | 1.116 | 0.042 | 0 | 1.114 | 0.060 | 0.000 | 1.116 | 0.054 | 0 | 1.115 | 0.054 | 0.000 | 1.114 | 0.060 | 0.00 |
| $\beta_{U}=$ 0, $\beta_{A}=$ 0.5 | 1.119 | 0.042 | 0 | 1.118 | 0.062 | 0.000 | 1.136 | 0.055 | 0 | 1.118 | 0.055 | 0.000 | 1.118 | 0.062 | 0.00 |
| $\beta_{U}=$ 0, $\beta_{A}=$ 1 | 1.118 | 0.042 | 0 | 1.116 | 0.066 | 0.000 | 1.174 | 0.058 | 0 | 1.117 | 0.058 | 0.000 | 1.116 | 0.066 | 0.00 |
| $\beta_{U}=$ -1, $\beta_{A}=$ 0 | 1.118 | 0.042 | 0 | 1.112 | 0.060 | 0.000 | 1.119 | 0.054 | 0 | 1.112 | 0.054 | 0.000 | 1.112 | 0.060 | 0.00 |
| $\beta_{U}=$ -1, $\beta_{A}=$ 0.1 | 1.119 | 0.042 | 0 | 1.118 | 0.059 | 0.000 | 1.121 | 0.053 | 0 | 1.117 | 0.053 | 0.000 | 1.118 | 0.059 | 0.00 |
| $\beta_{U}=$ -1, $\beta_{A}=$ 0.5 | 1.118 | 0.042 | 0 | 1.139 | 0.060 | 0.000 | 1.141 | 0.054 | 0 | 1.139 | 0.054 | 0.000 | 1.139 | 0.061 | 0.00 |
| $\beta_{U}=$ -1, $\beta_{A}=$ 1 | 1.117 | 0.042 | 0 | 1.157 | 0.064 | 0.000 | 1.182 | 0.057 | 0 | 1.156 | 0.057 | 0.000 | 1.157 | 0.064 | 0.00 |

*Table S7 (relates to Figure S1):* *Results for scenarios (iv) and (v), with $\gamma_{UA}=0.5$. Mean estimate of $\gamma_{A}$, width of confidence interval and coverage, for each simulation configuration (rows) and each missing data method (columns).*

|  | **Completed Data** | | | **Complete Case** | | | **MI(A)** | | | **MI(R+A)** | | | **MI(R*A)** | | |
| --- | --- | --- | --- | --- | --- | --- | --- | --- | --- | --- | --- | --- | --- | --- | --- |
| **Simulation Parameters** | **Est** | **Width** | **Cov** | **Est** | **Width** | **Cov** | **Est** | **Width** | **Cov** | **Est** | **Width** | **Cov** | **Est** | **Width** | **Cov** |
| $\beta_{U}=$ 1, $\beta_{A}=$ 0 | 1.251 | 0.045 | 0 | 1.190 | 0.063 | 0 | 1.224 | 0.056 | 0 | 1.221 | 0.056 | 0 | 1.190 | 0.064 | 0 |
| $\beta_{U}=$ 1, $\beta_{A}=$ 0.1 | 1.250 | 0.045 | 0 | 1.184 | 0.063 | 0 | 1.224 | 0.057 | 0 | 1.217 | 0.057 | 0 | 1.184 | 0.064 | 0 |
| $\beta_{U}=$ 1, $\beta_{A}=$ 0.5 | 1.252 | 0.045 | 0 | 1.171 | 0.063 | 0 | 1.242 | 0.056 | 0 | 1.211 | 0.056 | 0 | 1.171 | 0.065 | 0 |
| $\beta_{U}=$ 1, $\beta_{A}=$ 1 | 1.250 | 0.045 | 0 | 1.160 | 0.066 | 0 | 1.285 | 0.059 | 0 | 1.206 | 0.059 | 0 | 1.160 | 0.067 | 0 |
| $\beta_{U}=$ 0.5, $\beta_{A}=$ 0 | 1.251 | 0.045 | 0 | 1.219 | 0.063 | 0 | 1.235 | 0.056 | 0 | 1.235 | 0.056 | 0 | 1.219 | 0.064 | 0 |
| $\beta_{U}=$ 0.5, $\beta_{A}=$ 0.1 | 1.251 | 0.045 | 0 | 1.216 | 0.063 | 0 | 1.237 | 0.055 | 0 | 1.234 | 0.055 | 0 | 1.216 | 0.064 | 0 |
| $\beta_{U}=$ 0.5, $\beta_{A}=$ 0.5 | 1.250 | 0.045 | 0 | 1.207 | 0.064 | 0 | 1.258 | 0.056 | 0 | 1.233 | 0.057 | 0 | 1.207 | 0.064 | 0 |
| $\beta_{U}=$ 0.5, $\beta_{A}=$ 1 | 1.249 | 0.045 | 0 | 1.201 | 0.067 | 0 | 1.308 | 0.059 | 0 | 1.238 | 0.060 | 0 | 1.201 | 0.068 | 0 |
| $\beta_{U}=$ 0.1, $\beta_{A}=$ 0 | 1.249 | 0.045 | 0 | 1.243 | 0.064 | 0 | 1.246 | 0.056 | 0 | 1.246 | 0.056 | 0 | 1.243 | 0.064 | 0 |
| $\beta_{U}=$ 0.1, $\beta_{A}=$ 0.1 | 1.250 | 0.045 | 0 | 1.244 | 0.063 | 0 | 1.250 | 0.056 | 0 | 1.248 | 0.056 | 0 | 1.244 | 0.064 | 0 |
| $\beta_{U}=$ 0.1, $\beta_{A}=$ 0.5 | 1.251 | 0.045 | 0 | 1.242 | 0.064 | 0 | 1.276 | 0.057 | 0 | 1.257 | 0.057 | 0 | 1.242 | 0.064 | 0 |
| $\beta_{U}=$ 0.1, $\beta_{A}=$ 1 | 1.251 | 0.045 | 0 | 1.240 | 0.067 | 0 | 1.328 | 0.060 | 0 | 1.267 | 0.060 | 0 | 1.240 | 0.068 | 0 |
| $\beta_{U}=$ 0, $\beta_{A}=$ 0 | 1.250 | 0.045 | 0 | 1.250 | 0.063 | 0 | 1.250 | 0.056 | 0 | 1.250 | 0.056 | 0 | 1.250 | 0.063 | 0 |
| $\beta_{U}=$ 0, $\beta_{A}=$ 0.1 | 1.251 | 0.045 | 0 | 1.251 | 0.063 | 0 | 1.255 | 0.056 | 0 | 1.254 | 0.056 | 0 | 1.251 | 0.063 | 0 |
| $\beta_{U}=$ 0, $\beta_{A}=$ 0.5 | 1.251 | 0.045 | 0 | 1.251 | 0.064 | 0 | 1.280 | 0.056 | 0 | 1.263 | 0.056 | 0 | 1.251 | 0.064 | 0 |
| $\beta_{U}=$ 0, $\beta_{A}=$ 1 | 1.251 | 0.045 | 0 | 1.250 | 0.067 | 0 | 1.332 | 0.059 | 0 | 1.274 | 0.059 | 0 | 1.250 | 0.068 | 0 |
| $\beta_{U}=$ -1, $\beta_{A}=$ 0 | 1.250 | 0.045 | 0 | 1.310 | 0.063 | 0 | 1.284 | 0.056 | 0 | 1.281 | 0.056 | 0 | 1.310 | 0.062 | 0 |
| $\beta_{U}=$ -1, $\beta_{A}=$ 0.1 | 1.250 | 0.045 | 0 | 1.316 | 0.063 | 0 | 1.289 | 0.056 | 0 | 1.288 | 0.056 | 0 | 1.316 | 0.062 | 0 |
| $\beta_{U}=$ -1, $\beta_{A}=$ 0.5 | 1.251 | 0.045 | 0 | 1.334 | 0.063 | 0 | 1.323 | 0.056 | 0 | 1.318 | 0.056 | 0 | 1.334 | 0.063 | 0 |
| $\beta_{U}=$ -1, $\beta_{A}=$ 1 | 1.251 | 0.045 | 0 | 1.340 | 0.066 | 0 | 1.376 | 0.058 | 0 | 1.343 | 0.058 | 0 | 1.340 | 0.066 | 0 |

*Table S8 (relates to Figure S2):* *Results for scenario (vi), with $\gamma_{UA}=0.5$ and $\alpha_{U}=0.5$. Mean estimate of $\gamma_{A}$, width of confidence interval and coverage, for each simulation configuration (rows) and each missing data method (columns).*

|  | **Completed Data** | | | **Complete Case** | | | **MI(A)** | | | **MI(R+A)** | | | **MI(R*A)** | | |
| --- | --- | --- | --- | --- | --- | --- | --- | --- | --- | --- | --- | --- | --- | --- | --- |
| **Simulation Parameters** | **Est** | **Width** | **Cov** | **Est** | **Width** | **Cov** | **Est** | **Width** | **Cov** | **Est** | **Width** | **Cov** | **Est** | **Width** | **Cov** |
| $\beta_{U}=$ 1, $\beta_{A}=$ 0 | 1.368 | 0.043 | 0 | 1.304 | 0.061 | 0 | 1.337 | 0.055 | 0 | 1.330 | 0.055 | 0 | 1.304 | 0.062 | 0 |
| $\beta_{U}=$ 1, $\beta_{A}=$ 0.1 | 1.367 | 0.043 | 0 | 1.290 | 0.061 | 0 | 1.334 | 0.055 | 0 | 1.322 | 0.055 | 0 | 1.290 | 0.062 | 0 |
| $\beta_{U}=$ 1, $\beta_{A}=$ 0.5 | 1.367 | 0.043 | 0 | 1.255 | 0.062 | 0 | 1.347 | 0.056 | 0 | 1.302 | 0.056 | 0 | 1.255 | 0.063 | 0 |
| $\beta_{U}=$ 1, $\beta_{A}=$ 1 | 1.368 | 0.043 | 0 | 1.225 | 0.065 | 0 | 1.390 | 0.058 | 0 | 1.291 | 0.057 | 0 | 1.225 | 0.067 | 0 |
| $\beta_{U}=$ 0.5, $\beta_{A}=$ 0 | 1.368 | 0.043 | 0 | 1.337 | 0.061 | 0 | 1.352 | 0.055 | 0 | 1.350 | 0.055 | 0 | 1.337 | 0.062 | 0 |
| $\beta_{U}=$ 0.5, $\beta_{A}=$ 0.1 | 1.368 | 0.043 | 0 | 1.327 | 0.061 | 0 | 1.351 | 0.055 | 0 | 1.346 | 0.055 | 0 | 1.327 | 0.062 | 0 |
| $\beta_{U}=$ 0.5, $\beta_{A}=$ 0.5 | 1.369 | 0.043 | 0 | 1.298 | 0.062 | 0 | 1.366 | 0.056 | 0 | 1.334 | 0.056 | 0 | 1.298 | 0.063 | 0 |
| $\beta_{U}=$ 0.5, $\beta_{A}=$ 1 | 1.369 | 0.043 | 0 | 1.274 | 0.066 | 0 | 1.410 | 0.059 | 0 | 1.327 | 0.059 | 0 | 1.274 | 0.067 | 0 |
| $\beta_{U}=$ 0.1, $\beta_{A}=$ 0 | 1.367 | 0.043 | 0 | 1.361 | 0.061 | 0 | 1.364 | 0.055 | 0 | 1.364 | 0.055 | 0 | 1.361 | 0.061 | 0 |
| $\beta_{U}=$ 0.1, $\beta_{A}=$ 0.1 | 1.368 | 0.043 | 0 | 1.357 | 0.061 | 0 | 1.365 | 0.055 | 0 | 1.364 | 0.055 | 0 | 1.357 | 0.061 | 0 |
| $\beta_{U}=$ 0.1, $\beta_{A}=$ 0.5 | 1.367 | 0.043 | 0 | 1.330 | 0.062 | 0 | 1.378 | 0.055 | 0 | 1.356 | 0.055 | 0 | 1.330 | 0.063 | 0 |
| $\beta_{U}=$ 0.1, $\beta_{A}=$ 1 | 1.368 | 0.043 | 0 | 1.310 | 0.065 | 0 | 1.424 | 0.059 | 0 | 1.355 | 0.059 | 0 | 1.310 | 0.066 | 0 |
| $\beta_{U}=$ 0, $\beta_{A}=$ 0 | 1.369 | 0.043 | 0 | 1.369 | 0.061 | 0 | 1.368 | 0.054 | 0 | 1.368 | 0.054 | 0 | 1.369 | 0.061 | 0 |
| $\beta_{U}=$ 0, $\beta_{A}=$ 0.1 | 1.370 | 0.043 | 0 | 1.363 | 0.061 | 0 | 1.367 | 0.055 | 0 | 1.366 | 0.055 | 0 | 1.363 | 0.061 | 0 |
| $\beta_{U}=$ 0, $\beta_{A}=$ 0.5 | 1.368 | 0.043 | 0 | 1.341 | 0.062 | 0 | 1.384 | 0.055 | 0 | 1.364 | 0.055 | 0 | 1.341 | 0.062 | 0 |
| $\beta_{U}=$ 0, $\beta_{A}=$ 1 | 1.368 | 0.043 | 0 | 1.319 | 0.065 | 0 | 1.427 | 0.058 | 0 | 1.362 | 0.059 | 0 | 1.319 | 0.066 | 0 |
| $\beta_{U}=$ -1, $\beta_{A}=$ 0 | 1.369 | 0.043 | 0 | 1.421 | 0.061 | 0 | 1.403 | 0.054 | 0 | 1.396 | 0.054 | 0 | 1.421 | 0.060 | 0 |
| $\beta_{U}=$ -1, $\beta_{A}=$ 0.1 | 1.367 | 0.043 | 0 | 1.418 | 0.060 | 0 | 1.400 | 0.054 | 0 | 1.398 | 0.054 | 0 | 1.418 | 0.060 | 0 |
| $\beta_{U}=$ -1, $\beta_{A}=$ 0.5 | 1.366 | 0.043 | 0 | 1.412 | 0.060 | 0 | 1.414 | 0.054 | 0 | 1.410 | 0.054 | 0 | 1.412 | 0.060 | 0 |
| $\beta_{U}=$ -1, $\beta_{A}=$ 1 | 1.367 | 0.043 | 0 | 1.399 | 0.063 | 0 | 1.453 | 0.056 | 0 | 1.421 | 0.056 | 0 | 1.399 | 0.064 | 0 |

*Table S9 (relates to Figure S3):* *Results for scenario (vi-U), with $\gamma_{UA}=0$ and $\alpha_{U}=0.5$. Mean estimate of $\gamma_{A}$, width of confidence interval and coverage, for each simulation configuration (rows) and each missing data method (columns).*

|  | **Completed Data** | | | **Complete Case** | | | **MI(A)** | | | **MI(R+A)** | | | **MI(R*A)** | | |
| --- | --- | --- | --- | --- | --- | --- | --- | --- | --- | --- | --- | --- | --- | --- | --- |
| **Simulation Parameters** | **Est** | **Width** | **Cov** | **Est** | **Width** | **Cov** | **Est** | **Width** | **Cov** | **Est** | **Width** | **Cov** | **Est** | **Width** | **Cov** |
| $\beta_{U}=$ 1, $\beta_{A}=$ 0 | 1.000 | 0.055 | 0.965 | 1.000 | 0.070 | 0.945 | 0.999 | 0.064 | 0.955 | 0.999 | 0.064 | 0.955 | 1.000 | 0.066 | 0.955 |
| $\beta_{U}=$ 1, $\beta_{A}=$ 0.1 | 0.999 | 0.055 | 0.935 | 0.999 | 0.070 | 0.950 | 0.999 | 0.063 | 0.960 | 0.999 | 0.063 | 0.960 | 0.999 | 0.065 | 0.955 |
| $\beta_{U}=$ 1, $\beta_{A}=$ 0.5 | 0.999 | 0.055 | 0.945 | 0.999 | 0.071 | 0.950 | 1.012 | 0.064 | 0.860 | 0.999 | 0.064 | 0.955 | 0.999 | 0.066 | 0.935 |
| $\beta_{U}=$ 1, $\beta_{A}=$ 1 | 1.000 | 0.055 | 0.945 | 0.999 | 0.075 | 0.960 | 1.043 | 0.068 | 0.315 | 0.998 | 0.068 | 0.945 | 0.999 | 0.069 | 0.960 |
| $\beta_{U}=$ 0.5, $\beta_{A}=$ 0 | 1.000 | 0.055 | 0.940 | 1.001 | 0.074 | 0.940 | 0.999 | 0.065 | 0.970 | 0.999 | 0.065 | 0.970 | 1.000 | 0.068 | 0.955 |
| $\beta_{U}=$ 0.5, $\beta_{A}=$ 0.1 | 0.999 | 0.055 | 0.945 | 1.000 | 0.074 | 0.945 | 1.000 | 0.064 | 0.955 | 0.999 | 0.064 | 0.960 | 0.999 | 0.067 | 0.965 |
| $\beta_{U}=$ 0.5, $\beta_{A}=$ 0.5 | 1.000 | 0.055 | 0.965 | 0.999 | 0.074 | 0.950 | 1.014 | 0.066 | 0.875 | 1.000 | 0.066 | 0.950 | 1.000 | 0.069 | 0.970 |
| $\beta_{U}=$ 0.5, $\beta_{A}=$ 1 | 0.999 | 0.055 | 0.955 | 1.000 | 0.078 | 0.965 | 1.046 | 0.070 | 0.275 | 0.999 | 0.070 | 0.975 | 1.000 | 0.072 | 0.970 |
| $\beta_{U}=$ 0.1, $\beta_{A}=$ 0 | 1.002 | 0.056 | 0.945 | 1.002 | 0.078 | 0.915 | 1.001 | 0.067 | 0.960 | 1.001 | 0.067 | 0.960 | 1.002 | 0.071 | 0.960 |
| $\beta_{U}=$ 0.1, $\beta_{A}=$ 0.1 | 1.000 | 0.055 | 0.955 | 1.001 | 0.077 | 0.970 | 1.000 | 0.066 | 0.970 | 1.000 | 0.066 | 0.970 | 1.000 | 0.070 | 0.985 |
| $\beta_{U}=$ 0.1, $\beta_{A}=$ 0.5 | 1.000 | 0.055 | 0.940 | 1.000 | 0.078 | 0.975 | 1.014 | 0.067 | 0.880 | 0.999 | 0.066 | 0.975 | 1.000 | 0.070 | 0.985 |
| $\beta_{U}=$ 0.1, $\beta_{A}=$ 1 | 1.002 | 0.055 | 0.935 | 1.004 | 0.081 | 0.950 | 1.050 | 0.071 | 0.235 | 1.001 | 0.070 | 0.960 | 1.002 | 0.073 | 0.975 |
| $\beta_{U}=$ 0, $\beta_{A}=$ 0 | 1.000 | 0.055 | 0.945 | 1.000 | 0.078 | 0.940 | 1.000 | 0.066 | 0.965 | 1.000 | 0.066 | 0.965 | 1.001 | 0.071 | 0.955 |
| $\beta_{U}=$ 0, $\beta_{A}=$ 0.1 | 0.998 | 0.056 | 0.960 | 0.999 | 0.078 | 0.965 | 0.999 | 0.066 | 0.970 | 0.998 | 0.066 | 0.970 | 0.997 | 0.071 | 0.970 |
| $\beta_{U}=$ 0, $\beta_{A}=$ 0.5 | 1.001 | 0.055 | 0.910 | 1.001 | 0.078 | 0.920 | 1.014 | 0.068 | 0.880 | 1.000 | 0.068 | 0.935 | 1.000 | 0.071 | 0.950 |
| $\beta_{U}=$ 0, $\beta_{A}=$ 1 | 1.001 | 0.055 | 0.935 | 1.000 | 0.082 | 0.950 | 1.048 | 0.072 | 0.235 | 1.000 | 0.072 | 0.985 | 0.999 | 0.074 | 0.980 |
| $\beta_{U}=$ -1, $\beta_{A}=$ 0 | 1.001 | 0.055 | 0.945 | 1.003 | 0.090 | 0.940 | 1.000 | 0.069 | 0.975 | 1.000 | 0.069 | 0.975 | 1.002 | 0.079 | 0.985 |
| $\beta_{U}=$ -1, $\beta_{A}=$ 0.1 | 1.001 | 0.055 | 0.970 | 1.002 | 0.090 | 0.935 | 1.001 | 0.070 | 0.965 | 1.001 | 0.070 | 0.955 | 1.002 | 0.079 | 0.955 |
| $\beta_{U}=$ -1, $\beta_{A}=$ 0.5 | 1.000 | 0.055 | 0.925 | 1.002 | 0.089 | 0.955 | 1.014 | 0.071 | 0.920 | 1.000 | 0.071 | 0.975 | 1.001 | 0.078 | 0.965 |
| $\beta_{U}=$ -1, $\beta_{A}=$ 1 | 1.000 | 0.055 | 0.945 | 0.999 | 0.091 | 0.945 | 1.048 | 0.075 | 0.250 | 0.999 | 0.074 | 0.965 | 1.000 | 0.080 | 0.965 |
